# Spatial Distribution of the Cannabinoid Type 1 and Capsaicin Receptors May Contribute to the Complexity of Their Crosstalk

**DOI:** 10.1038/srep33307

**Published:** 2016-09-22

**Authors:** Jie Chen, Angelika Varga, Srikumaran Selvarajah, Agnes Jenes, Beatrix Dienes, Joao Sousa-Valente, Akos Kulik, Gabor Veress, Susan D. Brain, David Baker, Laszlo Urban, Ken Mackie, Istvan Nagy

**Affiliations:** 1 Section of Anaesthetics, Pain Medicine and Intensive Care, Department of Surgery and Cancer, Faculty of Medicine, Imperial College London, Chelsea and Westminster Hospital, 369 Fulham Road, London, SW10 9NH, UK; 2 Department of Anaesthesiology, Southwest Hospital, Third Military Medical University, Gaotanyan 19 Street, Shapingba, Chongqing 400038, P. R. China; 3 MTA-DE-NAP B-Pain Control Research GroupDepartment of Physiology, Faculty of Medicine, University of Debrecen, Nagyerdei krt. 98, Debrecen, H-4012, Hungary; 4 Institute of Physiology, University of Freiburg, Germany D-79104 Freiburg, Germany; 5 BIOSS Centre for Biological Signalling Studies, University of Freiburg, D-79104, Germany; 6 Department of Laboratory Medicine, Faculty of Medicine and Health, Örebro University, Örebro, Sweden; 7 BHF Cardiovascular Centre of Excellence and Centre of Integrative Biomedicine, Cardiovascular Division, King’s College London, London SE1 9NH, UK; 8 Centre for Neuroscience and Trauma, Blizard Institute, Barts and the London School of Medicine and Dentistry, Queen Mary University of London, 4 Newark Street, London, E1 2AT, UK; 9 Preclinical Secondary Pharmacology, Preclinical Safety, Novartis Institutes for Biommedical Research, Cambridge, MA 01932, USA; 10 Department of Psychological and Brain Sciences and Program in Neuroscience, Indiana University, The Gill Center, 702 N. Walnut Grove Avenue, Bloomington, IN 47405, USA

## Abstract

The cannabinoid type 1 (CB1) receptor and the capsaicin receptor (TRPV1) exhibit co-expression and complex, but largely unknown, functional interactions in a sub-population of primary sensory neurons (PSN). We report that PSN co-expressing CB1 receptor and TRPV1 form two distinct sub-populations based on their pharmacological properties, which could be due to the distribution pattern of the two receptors. Pharmacologically, neurons respond either only to capsaicin (COR neurons) or to both capsaicin and the endogenous TRPV1 and CB1 receptor ligand anandamide (ACR neurons). Blocking or deleting the CB1 receptor only reduces both anandamide- and capsaicin-evoked responses in ACR neurons. Deleting the CB1 receptor also reduces the proportion of ACR neurons without any effect on the overall number of capsaicin-responding cells. Regarding the distribution pattern of the two receptors, neurons express CB1 and TRPV1 receptors either isolated in low densities or in close proximity with medium/high densities. We suggest that spatial distribution of the CB1 receptor and TRPV1 contributes to the complexity of their functional interaction.

The capsaicin receptor, transient receptor potential cation channel, subfamily V, member 1 (TRPV1) is a non-selective cationic channel^1^. In addition to a series of exogenous molecules including capsaicin the pungent agent of chili peppers, TRPV1 is also directly activated, among other endogenous agents, by N-arachidonoylethanolamine (anandamide)^2^,^3^,^4^. Anandamide is also an endogenous ligand for the G protein-coupled cannabinoid (CB) type 1 receptor^2^.

Nociceptive primary sensory neurons (PSN) constitute the prototypical cell type, which expresses TRPV1^1^,^5^. Activation of TRPV1 results in cationic influx, subsequent depolarisation and action potential generation^1^,^4^. The CB1 receptor, reduces neuronal excitability^6^,^7^ through the activation of the G protein-coupled inwardly rectifying K^+^ channel and the inhibition of adenylyl cyclase and high voltage-activated Ca_v_1.2, Ca_v_2.1, and Ca_v_2.2 Ca^2+^ channels conducting L-type, P/Q-type, and N-type currents, respectively^8^,^9^,^10^. CB1 receptor activation may also increase neuronal excitation through coupling to Gs or Gq/11, and the activation of adenylyl cyclase, phosphatidylinositol-3 kinase, and the mitogen-activated kinases, extracellular signal-regulated kinase 1 and 2, and p38^8^,^9^,^10^.

The CB1 receptor and TRPV1 exhibit co-expression in various neurons including a major sub-population of PSN in dorsal root ganglia (DRG^11^,^12^,^13^ but see^14^,^15^). The anatomical arrangement between these two receptors allows a complex, but currently largely unknown, crosstalk that involves activation of both TRPV1 and the CB1 receptor by anandamide.

In order to better understand how the CB1 – TRPV1 crosstalk shapes neuronal excitability, we herein investigated the functional interaction between the receptors, with particular attention to their spatial distribution in PSN and the effect of anandamide.

## Results

**Figure 1:**
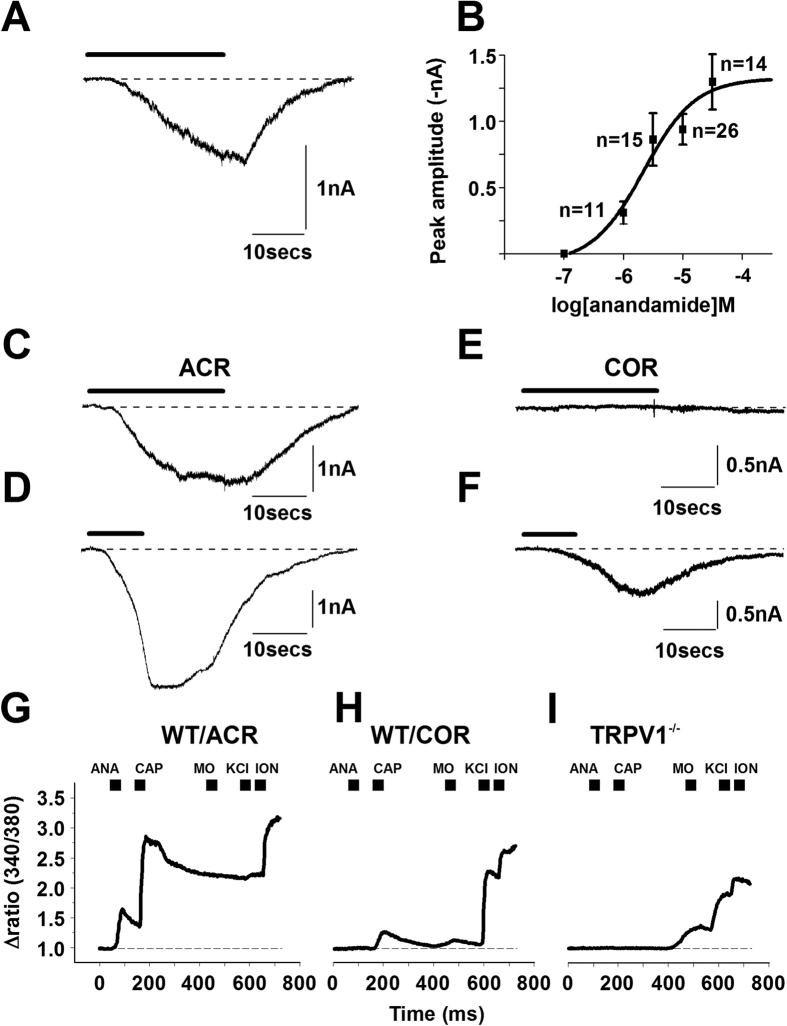
Anandamide- and capsaicin-evoked responses are partially segregated in cultured primary sensory neurons. (**A**) A typical response to anandamide (10 μM) recorded from cultured rat PSN by conventional whole-cell voltage-clamp. A sub-population of cells responds to anandamide. The response is washed off within a few tens of seconds after stopping anandamide application, which is indicated by the bar above the current trace. (**B**) Concentration-response relationship of anandamide in cultured rat PSN (data were fitted with Hill’s equation: y = Vmax*x^n/(k^n + x^n)). The calculated EC_50_ is 2.2 μM; the maximal effect was reached at 30 μM. (**C**,**D**) A typical whole-cell current from a cultured rat PSN during anandamide (bar, 30 μM) application. Capsaicin-evoked response from the same neuron (**D**). Bar indicates capsaicin (500 nM) application. A group of cultured PSN, which are regarded as “anandamide- and capsaicin-responsive” (ACR) neurons, exhibits this response pattern. (**E**,**F**) About a third of the rat PSNs are capsaicin-only-responsive neurons (COR). Typical whole-cell voltage-clamp recording from a COR neuron, which fails to respond to anandamide (E; bar, 30 μM) but exhibits a response to 500 nM capsaicin (**F**). Bar indicates capsaicin application. (**G**) Changes in the intracellular Ca^2+^ concentrations were measured by a conventional ratiometric approach in cultured PSN isolated from wild type (WT) or TRPV1 knock out (TRPV1^−/−^) mice following the application of 30 μM anandamide (ANA), 500 nM capsaicin (CAP), 30 μM mustard oil (MO), 50 mM KCl and 5 μM ionomycin (ION). A typical recording of Ca^2+^ transients from a PSN which is isolated from a WT mouse and responds to both anandamide and capsaicin. (**H**) A typical recording of Ca^2+^ transients from a neuron which is isolated from a WT mouse and responds to capsaicin but not to anandamide. (**I**) Cells isolated from TRPV1^−/−^ mice do not respond to either anandamide or capsaicin, however a sub-population of these neurons responds to mustard oil.

Anandamide applied for 20 seconds at a concentration range (1 μM, 3 μM, 10 μM and 30 μM) known to induce excitation^3^,^16^ of cultured rat PSN, produced concentration-dependent inward currents at −60 mV membrane potential with an EC_50_ of 2.2 μM (Fig. 1A,B; Supplementary Table 1). The excitatory effect of anandamide was washed off within a few tens of seconds after stopping anandamide application (Fig. 1A,C). Anandamide at 30 μM reached its maximal excitatory effect (Fig. 1B).

All the anandamide-responding neurons that were tested for capsaicin-responsiveness (Supplementary Table 2) produced currents to capsaicin applied at around its EC_50_ value (500 nM^1^,^4^), 2 minutes after anandamide superfusion (n = 29; Fig. 1C,D). However, a sub-population of neurons, while not responding to anandamide responded only to capsaicin (n = 10; Fig. 1E,F; Table 1 and Supplementary Table 2). Hence, anandamide- and capsaicin-responsiveness defined two sub-populations of PSN: the “anandamide-and-capsaicin-responsive” (ACR) and the “capsaicin-only-responsive” (COR) neurons (Fig. 1C–F).

The proportion of COR neurons in the overall sample of capsaicin-responsive cells was 25.6% (10 of the 39 cells; Table 1; Supplementary Table 2) and independent of anandamide concentration up to 30 μM (Supplementary Table 2; p = between 1 and 0.6, Fisher’s exact test). The concentration of anandamide, applied before capsaicin application, did not have significant effect on the amplitude of capsaicin-evoked responses in ACR or COR neurons (data not shown; p = between 0.98 and 0.11, ANOVA). Therefore, we pooled the amplitudes of capsaicin-evoked responses in ACR and COR neurons, respectively. The pooled capsaicin-evoked amplitudes were significantly different (ACR: −3.19 ± 0.31 nA, n = 29; COR: −0.96 ± 0.27 nA, n = 10; p = 0.002, Student’s t-test).

Since the anandamide concentration had no effect on either the proportion of responsive cells or the response to subsequent capsaicin application, we used anandamide at 30 μM in the rest of the experiments.

TRPV1 is highly permeable to Ca^2+ ^1,4. Therefore, to confirm the presence of ACR and COR neurons, we studied changes in the intracellular Ca^2+^ concentration ([Ca^2+^]_i_) by anandamide (30 μM) and capsaicin (500 nM) in rat cultured PSN grown for 16–22 hours.

Of the 98 KCl-responding neurons, 57 responded to capsaicin. Only 40 of the capsaicin-responsive neurons responded also to anandamide (Supplementary Figure 1). The ACR:COR neuron ratio was not significantly different from that found using whole-cell recordings (Table 1). The amplitude of the capsaicin-evoked calcium transients was significantly greater in ACR than in COR neurons (1.01 ± 0.12 in ACR (n = 40) and 0.49 ± 0.13 (n = 17); Supplementary Figure 1; p = 0.015, Student’s t-test). The TRPV1 antagonist capsazepine (10 μM) blocked the anandamide-evoked Ca^2+^ transients indicating that the anandamide-evoked excitatory effect is indeed mediated through TRPV1 in PSN (Supplementary Figure 2).

Next we assessed 30 μM anandamide- and 500 nM capsaicin-evoked changes in [Ca^2+^]_i_ in PSN cultures prepared from WT-TRPV1 and TRPV1^−/−^ mouse DRG (Fig. 1G–I) to determine whether ACR and COR neurons are also present in another species and the excitatory effects by both anandamide and capsaicin are indeed mediated exclusively through TRPV1. To verify chemosensitivity of PSN isolated from TRPV1^−/−^ mice, we also administered mustard oil (50 μM) following capsaicin application to the cells.

Responses of 136 KCl-responding WT-TRPV1 cells were analysed. Of these, 54 neurons responded to capsaicin, of which 37 were ACR and 17 were COR neurons (Fig. 1G,H; Table 1). The ACR:COR ratio was not significantly different from that found with whole-cell recordings or Ca^2+^ imaging from rat cultured PSN (Table 1). The increase in [Ca^2+^]_i_ induced by capsaicin was significantly smaller in COR neurons than in ACR cells (ACR: 2.09 ± 0.13 (n = 37); COR: 0.81 ± 0.17 (n = 17); p < 0.0001, Student’s t-test).

Neither anandamide nor capsaicin increased the [Ca^2+^]_i_ in the 126 TRPV1^−/−^ neurons we assessed (Fig. 1I). However, 36 of the 126 neurons responded to mustard oil indicating that in general, chemosensitivity of the neurons remained intact (Fig. 1I).

These data show that anandamide- and capsaicin-responsiveness are partially segregated in rodent PSN. Although we cannot categorically exclude the possibility that compensatory changes in TRPV1^−/−^ mice contribute to the lack of anandamide responsiveness, we suggest that both the anandamide- and capsaicin-evoked excitation depend on TRPV1 in PSN. Considering that TRPV1 mediates the excitatory effect of both capsaicin and anandamide, the behaviour of ACR and COR neurons is underlain by differential responsiveness of TRPV1 to these agents in the two types of neurons.

The two main sub-populations of nociceptive PSN, the peptidergic and *Bandeiraea simplicifolia* lectin (IB4)-binding cells^17^, exhibit different capsaicin-evoked responses^18^,^19^. However, our data indicate that the presence or absence of neuropeptides do not contribute to the partial segregation of anandamide- and capsaicin-responsiveness in PSN (Supplementary Table 3).

The CB1 receptor is able to regulate TRPV1 responsiveness^16^,^20^,^21^,^22^,^23^,^24^,^25^,^26^,^27^. Further, rimonabant at less than ~200 nM is a selective and specific CB1 receptor antagonist/inverse agonist^28^,^29^. Therefore, we used 200 nM rimonabant to determine whether the CB1 receptor contributes to the segregation of responses.

**Figure 2:**
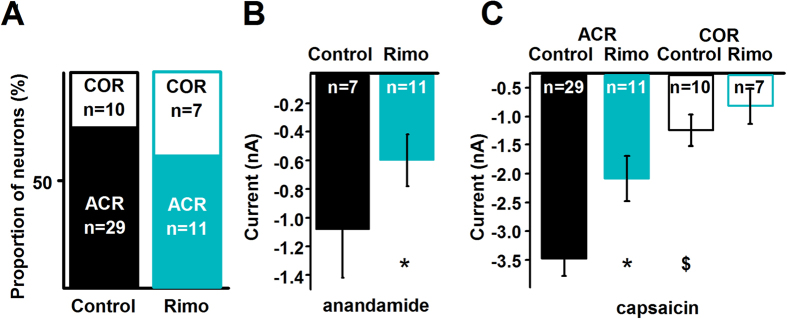
Rimonabant reduces the amplitude of anandamide- and capsaicin-evoked currents only in ACR neurons without affecting the ratio of ACR and COR cells. (**A**) Whole-cell currents evoked by 30 μM anandamide and subsequent 500 nM capsaicin applications were also recorded when 200 nM rimonabant (Rimo), a highly selective and specific antagonist/inverse agonist of the CB1 receptor was continuously applied to the cells through the bath solution in order to find the effect of the CB1 receptor on anandamide- and capsaicin-evoked responses in ACR and COR neurons. The presence of rimonabant in the bath solution does not change the ratio of ACR and COR neurons (p = 1, Fisher’s exact test). (**B**) Average maximum amplitudes of anandamide-evoked currents produced by cultured rat PSN in the control medium and in the presence of 200 nM rimonabant (Rimo). Rimonabant significantly reduces the amplitude of anandamide-evoked currents (p = 0.006, Student’s t-test). (**C**) Average maximum amplitudes of capsaicin-evoked currents produced by ACR and COR type cultured rat PSN in control medium and in the presence of 200 nM rimonabant (Rimo). The average maximum amplitude of the capsaicin-evoked currents in COR type neurons is significantly smaller (^$^p = 0.002, Student’s t-test) than that in ACR type neurons in the control bath solution. While the presence of rimonabant significantly reduces the average maximum amplitude of capsaicin-evoked currents in ACR neurons (*p = 0.001, Student’s t-test), it has no significant effect on the average maximum amplitude of capsaicin-evoked currents in COR neurons (p = 0.34, Student’s t-test).

First we used whole-cell voltage-clamp recordings from cultured rat PSN to facilitate comparison with data obtained in the first experiment (Table 1). Rimonabant, which was continuously superfused, did not change the ACR:COR neuron ratio (Fig. 2A; Table 1). However, interpretation of this finding requires caution due to the relatively low number of neurons. Nevertheless, the amplitude of 30 μM anandamide-evoked currents was significantly reduced from −1.07 ± 0.33 nA (n = 7) to −0.59 ± 0.18 nA (n = 11; Fig. 2B; p = 0.006, Student’s t-test; Supplementary Table 2) with rimonabant. Rimonabant significantly reduced the amplitude of the capsaicin-evoked responses in ACR neurons (from −3.19 ± 0.31 nA (n = 29) to −1.82 ± 0.40 nA (n = 11); p < 0.001, Student’s t-test) but not in COR neurons (−0.96 ± 0.27 nA (n = 10) to −0.54 ± 0.31 nA (n = 7), p = 0.34, Student’s t-test; Fig. 2C). Because of the high variability of data on COR neurons, interpretation requires caution. Nevertheless, these findings did suggest that the CB1 receptor might have a sensitising effect on capsaicin-evoked responses in ACR neurons, which might not be present in COR neurons. Subsequently, we conducted experiments to confirm the CB1-mediated sensitising effect on TRPV1 and to study the underlying mechanisms.

First, to confirm the role of the CB1 receptor in sensitising TRPV1 in ACR neurons, we isolated PSN from Biozzi ABH (WT-CB1) and Biozzi ABH mice lacking the CB1 receptor (CB1^−/−^). We have chosen these mice, because they exhibit a significantly altered endocannabinoid system in comparison to C57BL/6 mice, and we used Biozzi ABH mice in previous studies^30^. Due to the significantly altered endocannabinoid system, we expected to find differences in TRPV1-mediated responses in C57BL/6 (background for TRPV1^−/−^ mice) and Biozzi ABH mice if our hypothesis on the sensitising effect of the CB1 receptor on TRPV1 were correct. To increase the efficacy of sampling, we again assessed changes in the [Ca^2+^]_i_.

**Figure 3:**
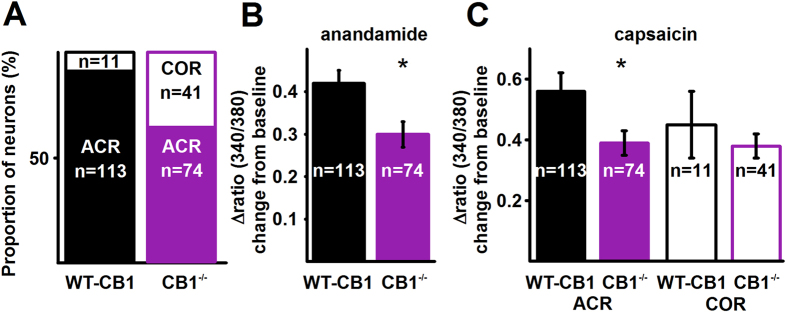
Deletion of the CB1 receptor reduces both anandamide- and capsaicin-evoked responses in ACR neurons and changes the ACR – COR ratio. (**A**) Conventional ratiometric approach was used to find the effect of deleting the CB1 receptor in Biozzi ABH mice on anandamide- and capsaicin-evoked calcium transients in ACR and COR type cultured PSN. The ratio of ACR and COR neurons in cultures prepared from wild type (WT-CB1) and CB1 receptor knock out (CB1^−/−^) mice. Deletion of the CB1 receptor significantly reduces the number of ACR neurons (p < 0.0001, Fisher’s exact test), whereas it significantly increases the number of COR neurons (p < 0.0001, Fisher’s exact test). The overall number of capsaicin-sensitive cells (ACR + COR) however is not changed significantly (p = 0.56, Fischer’s exact test, see Results). (**B**) Average amplitudes of anandamide-evoked calcium transients in ACR neurons collected from wild type (WT-CB1) and CB1 receptor knock out (CB1^−/−^) mice. Deletion of the CB1 receptor results in a significant reduction in anandamide-evoked calcium transients (p = 0.02, Student’s t-test). (**C**) Average amplitudes of capsaicin-evoked calcium transients in ACR and COR neurons collected from wild type (WT-CB1) and CB1 receptor knock out (CB1^−/−^) mice. Deletion of the CB1 receptor results in significantly reduced amplitude of capsaicin-evoked calcium transients in ACR (p = 0.01, Student’s t-test) but not in COR neurons (p = 0.42, Student’s t-test).

Anandamide increased the [Ca^2+^]_i_ in 113 of 230 KCl-responding neurons of WT-CB1 mice, all of which responded to capsaicin (Fig. 3A). In addition to the 113 ACR neurons, 11 of the 230 neurons exhibited responses only to capsaicin (Fig. 3A). The ACR:COR neuron ratio was significantly higher in WT-CB1 mice than in WT-TRPV1 mice or rats (Table 1). These data supports our view that the cannabinoid system contributes to regulating TRPV1 responsiveness. To get direct evidence for the involvement of the CB1 receptor, we next measured responses in PSN cultures prepared from CB1^−/−^ mice.

The proportion of neurons responding to anandamide was significantly reduced within the total number of capsaicin-responding cells in CB1^−/−^ mice (Fig. 3A; Table 1). At the same time, the proportion of COR neurons within the total number of capsaicin-responding neurons was significantly increased (Fig. 3A; Table 1). However, the overall proportion of neurons responding to 500 nM capsaicin in CB1^−/−^ mice (115 of 200) was not statistically different to that in WT-CB1 mice (124 of 230, p = 0.56, Fischer’s exact test).

Deleting the CB1 receptor significantly reduced the amplitude of both anandamide (from 0.42 ± 0.03, n = 113, to 0.30 ± 0.03, n = 74, p = 0.02, Student’s t-test; Fig. 3B) and capsaicin-evoked responses (from 0.56 ± 0.06, n = 113, to 0.39 ± 0.04, n = 74, p = 0.01, Student’s t-test; Fig. 3C) in ACR neurons. However, there was no significant difference in the amplitude of capsaicin-evoked responses between COR neurons isolated from WT-CB1 and CB1^−/−^ mice (from 0.45 ± 0.11, n = 11, to 0.38 ± 0.04, n = 41, p = 0.42, Student’s t-test; Fig. 3C). These findings confirm that the CB1 receptor executes a sensitising effect on TRPV1, which increases capsaicin- and anandamide-evoked responses in ACR neurons. Further, these findings also indicate that in addition to sensitising TRPV1, the CB1 receptor also contributes to the partial segregation of anandamide- and capsaicin-responsiveness in PSN.

CB1 receptor activity can be evoked by endogenous ligands, such as anandamide, synthesised by a major proportion of TRPV1-expressing (hence CB1 receptor-expressing^11^,^12^) PSN^27^,^31^,^32^,^33^. Alternatively, the CB1 receptor-mediated effects could be due to its constitutive activity^28^,^34^. To differentiate between these mechanisms, we next compared the effects on capsaicin-evoked responses of rimonabant, which blocks both evoked and constitutive activity, and NESS0327^35^, which only inhibits ligand binding. To exclude the possibility of CB1 receptor activation by exogenous anandamide, capsaicin application was not preceded by anandamide superfusion. We assessed changes in the [Ca^2+^]_i_ to increase efficacy and we applied 100 nM capsaicin to diminish TRPV1 desensitisation^36^.

**Figure 4:**
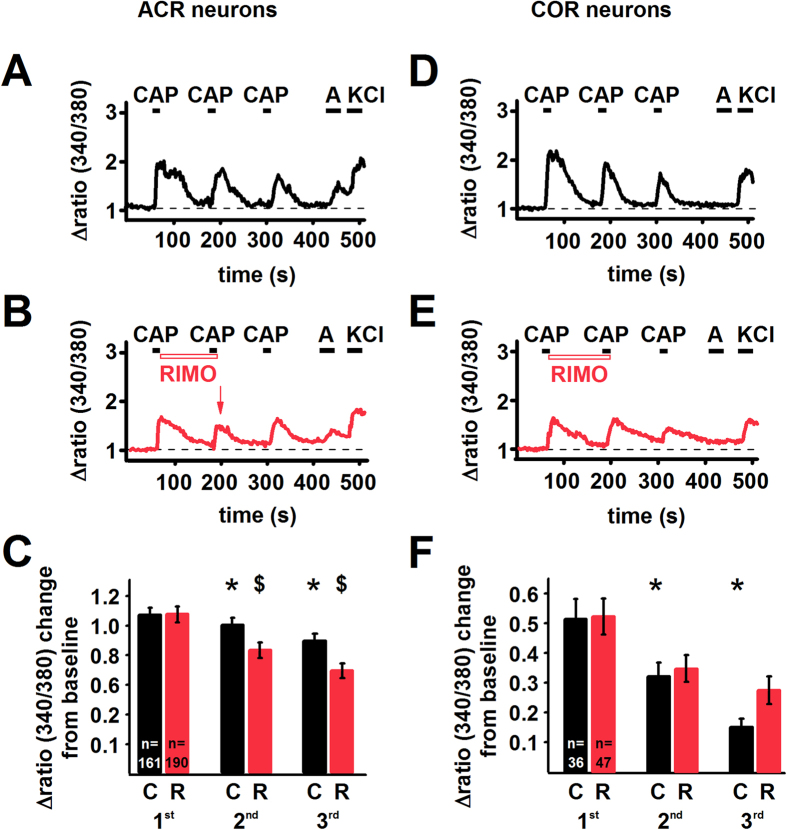
Capsaicin-evoked Ca^2+^ transients are significantly reduced by rimonabant in ACR but not in COR cultured rat PSN. (**A**) Conventional ratiometric approach was used to find the effect of the CB1 receptor antagonist/inverse agonist rimonabant (200 nM; RIMO) on capsaicin-evoked calcium transients in cultured rat PSN. (A) 100 nM capsaicin (CAP) was applied in every 2 minutes followed by 30 μM anandamide (**A**) and 50 mM KCl. The recording shows calcium transients evoked by consecutive capsaicin application in an ACR type neuron in a control experiment when no rimonabant was applied. Capsaicin induces the typical desensitisation. (**B**) During the recording from another ACR type neuron, rimonabant (RIMO) was applied immediately after the first capsaicin application until the end of the second capsaicin application. Rimonabant reduces the amplitude of the Ca^2+^ transient evoked by the second capsaicin application (arrow). In this neuron, the capsaicin-evoked response exhibits partial recovery following the removal of rimonabant. (**C**) Average amplitudes of calcium transients evoked by consecutive application of 100 nM capsaicin in every 2 minutes in ACR type neurons either without (C; black bars) or with the application of 200 nM rimonabant (R; red bars). In control experiments capsaicin induces significant desensitisation (*p < 0.0001, Student’s t-test both for the second and third responses). Rimonabant produces a significant reduction in the amplitude of the calcium transients evoked by the second (^$^p = 0.0001, Student’s t-test) and third capsaicin (^$^p < 0.0001, Student’s t-test) applications. (**D**) Calcium transients evoked by consecutive capsaicin application in a COR type neuron (note the lack of response to anandamide). (**E**) Rimonabant (RIMO) has no effect on the amplitude of the Ca^2+^ transient evoked by the second capsaicin application in a COR neuron. (**F**) Average amplitudes of calcium transients evoked by consecutive application of 100 nM capsaicin in every 2 minutes in COR type neurons either without (C; black bars) or with the application of 200 nM rimonabant (R; red bars). In control experiments capsaicin induced desensitisation (*p < 0.0001, Student’s t-test). Rimonabant does not produce any significant change in the amplitude of the calcium transients evoked by the capsaicin applications.

Capsaicin was applied 3 times consecutively with 2 minutes intervals to identify capsaicin responsive cells. The further application of 30 μM anandamide revealed 161 ACR and 36 COR neurons in rat cultured PSN (Fig. 4; Table 1). This ratio was not significantly different from our previous findings with either whole-cell recordings or Ca^2+^ imaging from cultured rat PSN (Table 1). The average amplitudes of the first capsaicin-evoked responses in ACR neurons (1.07 ± 0.05, n = 161) was significantly greater than that in COR neurons (0.51 ± 0.07, n = 36; p < 0.0001, Student’s t-test; Fig. 4C,F).

100 nM capsaicin induced desensitisation in both ACR and COR neurons (Fig. 4A,C,D,F). Interestingly, the desensitisation was significantly greater in COR than in ACR neurons (Fig. 4A,C,D,F).

While rimonabant, applied immediately after the first capsaicin application until the end of the second capsaicin application (Fig. 4B,E), did not have any effect on the [Ca^2+^]_i_ *per se*, it significantly increased the rate of reduction in the average amplitude of the second capsaicin-evoked responses in ACR (Fig. 4B,C; p = 0.0001, Student’s t-test) but not in COR neurons (Fig. 4E,F; p = 0.62, Student’s t-test). Although, several ACR neurons exhibited partial recovery in their capsaicin-evoked responses after stopping rimonabant application (Fig. 4B), the rate of reduction in the average amplitude of the third capsaicin-evoked responses in these cells was still significantly greater when compared to that of the control experiment (Fig. 4C; p < 0.0001, Student’s t-test). No such differences were seen in COR neurons (Fig. 4F, p = 0.084; Student’s t-test). Notably, in addition to reducing the amplitude of the capsaicin-evoked responses in ACR cells, rimonabant also prolonged the recovery of responses in a group of both types of neurons (Supplementary Figure 3).

In contrast to rimonabant, NESS0327 at 100 nM *per se* substantially enhanced the duration of the capsaicin-evoked Ca^2+^ transients (Supplementary Figure 4A,B). This increase did not allow further assessment in the great majority of cells (Supplementary Figure 4A,B). The [Ca^2+^]_i_-increasing effect was less pronounced at 1 nM, and when NESS0327 was applied 30 seconds after stopping capsaicin application an apparently capsaicin-independent increase in the [Ca^2+^]_i_ was produced (Supplementary Figure 4C). In this condition, 1 nM NESS0327 did not produce any effect on the amplitude of the second capsaicin application-evoked responses in COR neurons (n = 9; p = 0.74, Student’s t-test), whereas an increase was apparent in ACR neurons (n = 25; control: 94.66 ± 1.74% of the first response; NESS0327: 116.76 ± 9.52% of the first response; p = 0.0002, Student’s t-test; Supplementary Figure 4D,E). Although the excitatory effect of NESS0327 on PSN, the elucidation of which was not in the scope of the present study, makes interpretation of these findings very difficult, the lack of inhibitory effect may suggest that ligand-induced activation of the CB1 receptor does not contribute to TRPV1 sensitisation in PSN.

The finding that CB1 receptor inhibition or deletion has no effect on COR neurons may suggest that COR neurons might not express the CB1 receptor. To test this hypothesis, we next studied CB1 receptor expression in PSN by single-cell reverse transcription polymerase chain reaction (RT-PCR).

**Figure 5:**
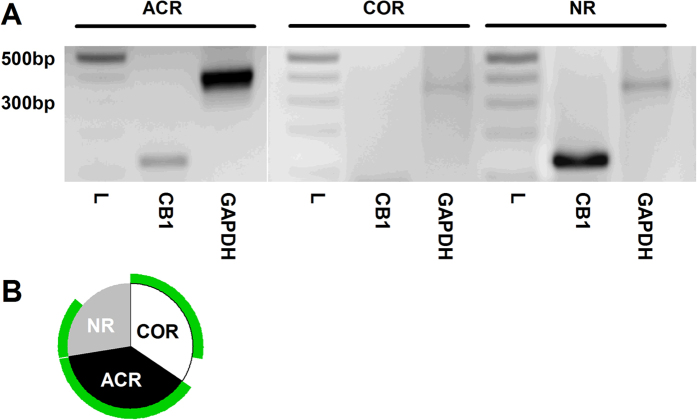
The CB1 receptor is expressed by all ACR type and the majority of COR type cultured rat cultured PSN. (**A**) After determining the types of cultured rat PSN by whole-cell voltage-clamp recordings by anandamide (30 μM) and capsaicin (500 nM) application, 76 cells were collected for single-cell PCR. 10 COR type, 11 ACR type and 8 “anandamide- and capsaicin non-responding” neurons (NR) were successfully processed to find out whether the neuron expressed the CB1 receptor. The sizes of the amplicons generated to specific primers for CB1 receptor (127 bp) or the house-keeping molecule GAPDH (380 bp) were indistinguishable from the predicted sizes as it is shown by these typical gel images. L indicates the size marker. Note the presence of the GAPDH and the lack of CB1 amplicon in a COR neuron. (**B**) All the ACR cells and 8 of 10 COR neurons express the CB1 receptor in this sample of neurons. In addition 50% of the non-responding cells also express the CB1 receptor.

We collected 25 COR, 31 ACR and 20 cells, which did not respond to either of the TRPV1 activators (NR) from rat PSN cultures. The ACR:COR neuron ratio was not significantly different from the observation we made in whole-cell recordings from cultured rat PSN (Table 1). The amplitude of capsaicin-evoked responses was significantly larger (p < 0.0001, Student’s t-test) in ACR (−3.28 ± 0.35 nA, n = 31) than in COR (−0.67 ± 0.06 nA, n = 25) neurons. RNA of 10 COR, 11 ACR and 8 NR cells were processed successfully with single-cell RT-PCR (i.e. the sample showed GAPDH mRNA expression; Fig. 5A,B).

CB1 receptor primers produced amplicons of the predicted size, whereas no amplicons were present when no template was included in the PCR mixture (Fig. 5A; Supplementary Figure 5). All ACR neurons (n = 11), eight of ten COR neurons, and four of eight NR neurons exhibited detectable CB1 receptor mRNA expression (Fig. 5B). Immunolabelling of cultured rat PSN using an anti-CB1 receptor and anti-TRPV1 antibody confirmed the high proportion of TRPV1-expressing cells also expressing the CB1 receptor (149 of 150, Table 1; Supplementary Figure 6).

For the control of immunostaining and assessing possible differences between the intact DRG and cultured PSN, we also established the co-expression ratio of TRPV1 and the CB1 receptor in the intact DRG (Supplementary Figures 7 and 8). We found a slightly smaller proportion of TRPV1-expressing cells exhibiting obvious immunopositivity for the CB1 receptor in intact DRG than in PSN cultures (88.3 ± 3.8%, n = 3). This small difference could be due to culturing. Nevertheless, together, these findings indicate that the differing effects of rimonabant or deleting the CB1 receptor in ACR and COR neurons cannot be due to the exclusive expression of the CB1 receptor in ACR type neurons. Therefore, next we assessed the spatial distribution pattern of the CB1 receptor and TRPV1.

**Figure 6:**
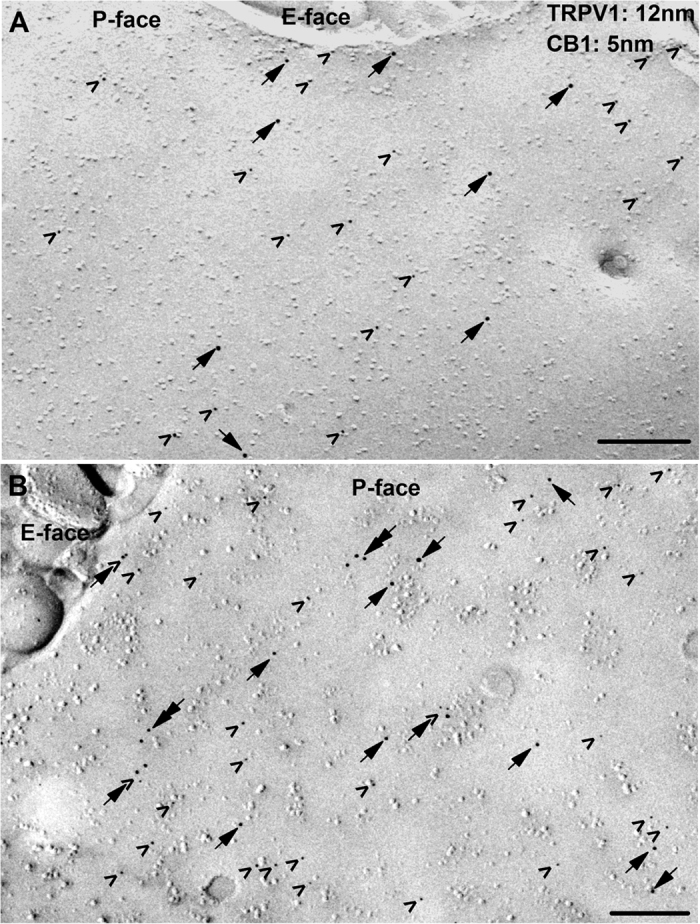
The SDS-FRL method reveals two major types of CB1 receptor and TRPV1 spatial distribution in DRG neurons. (**A**,**B**) Double immunogold labelling for TRPV1 (12 nm particles; arrows) and the CB1 receptor (5 nm; arrowheads) reveals that the immunoreactivity for both proteins is moderate (**A**) to strong (**B**) on the protoplasmic face (P-face) but not on the exoplasmic face (E-face) of plasma membrane of PSN. In some patches of putative somatic membrane of PSN, the great majority of immunoparticles for TRPV1 and CB1 are isolated from each other (**A**), whereas on other patches of the membrane immunoparticles for TRPV1 and CB1 form co-clusters (arrow + arrowhead in B). Note the occasional co-clustering of particles labelling TRPV1 (double arrows in B). Scale bars = 0.2 μm.

Immunocytochemistry on SDS-digested freeze-fracture replicas (SDS-FRL) is a highly effective method to reveal sub-cellular protein distribution^37^. TRPV1-labelling, which served as a basis for selection of putative somatic membranes of PSN, was generally moderate to strong (Fig. 6A,B). All TRPV1-expressing membranes we examined exhibited immunolabelling for the CB1 receptor, which was also moderate to strong in general. Both TRPV1 and CB1 receptor immunoreactivities were specifically detected on the protoplasmic face (Fig. 6A,B). Virtually no labelling was observed on the exoplasmic face (Fig. 6A,B), which is in agreement with the epitope of both the anti-TRPV1- and anti-CB1 receptor antibodies raised against intracellular part of the respective molecules (see Material and Methods).

Membrane patches co-expressing TRPV1 and CB1 receptors differed in receptor densities and distribution (Fig. 6A,B). Some of the patches exhibited only a few isolated receptors (Fig. 6A). The majority of the membrane patches however exhibited a relatively high density of CB1 receptor expression, which was accompanied by moderate to high TRPV1 receptor density (Fig. 6B). It is unlikely that the differences in densities could be due to major regional variation of receptor distribution within a single cell, because no such differences were evident by visual assessment of about 100 TRPV1 and CB1 receptor co-expressing cultured PSN neurons or PSN in intact DRG (Supplementary Figure 6 and Supplementary Figure 8).

We found that two, three or sometimes four immunoparticles labelling TRPV1 in these membranes often formed clusters (Fig. 6B). The distance of the gold particles in these clusters was between ~10 and ~55 nm (Fig. 6B). Although the majority of the CB1 receptors appeared solitary, some of them seemed to be in close vicinity (<~55 nm) to TRPV1 (Fig. 6B).

We established morphometric data of 21 membrane patches (Table 2), which had an average size of 5.20 ± 0.39 μm^2^ and exhibited 2788 gold particles of which 1793 (85.38 ± 16.03, n = 21, on average) and 995 (47.38 ± 6.78, n = 21, on average) labelled CB1 receptor and TRPV1, respectively. The densities for both receptors varied in a wide range (Table 2) and the CB1 receptor density was significantly higher than that of TRPV1 (p = 0.024, Student’s t-test). Correlations between some properties are shown in Supplementary Figure 9.

We also established the number of distances measured between each TRPV1 and its closest CB1 receptor neighbour as well as between each CB1 receptor and its closest TRPV1 neighbour, which were below 54.5 nm (Table 2). This threshold represents the maximum (“critical”) distance between the centres of the gold particles if the two receptors are positioned next to each other, and is based on the estimated size of the CB1 receptor, TRPV1, IgG and the gold particles^38^,^39^,^40^,^41^ (Table 2; Supplementary Figure 10A). The number of distances between each TRPV1 and the closest TRPV1 neighbour shorter than 56 nm, which represents the estimated maximum distance if TRPV1 molecules are located next to each other (Supplementary Figure 10B), was also established. The number of critical distances was then divided by the number the appropriate receptor in each patch.

Principal component analysis (PCA), an unsupervised multivariate statistical tool, which reduces the number of variables and maximises the variability of data, suggested the presence of 2 major groups and 3 outliers (Supplementary Figure 11). PCA’s loading plot suggested that the number of CB1 receptor – TRPV1 critical distances does not contribute significantly to the variability of data (not shown). After removing the outliers and non-significant variables, PCA identified three principal components (PCs) that together contributed to more than 80% of the variability. The PCA score plot suggested the presence of 2–4 groups of cells (Supplementary Figure 11).

**Figure 7:**
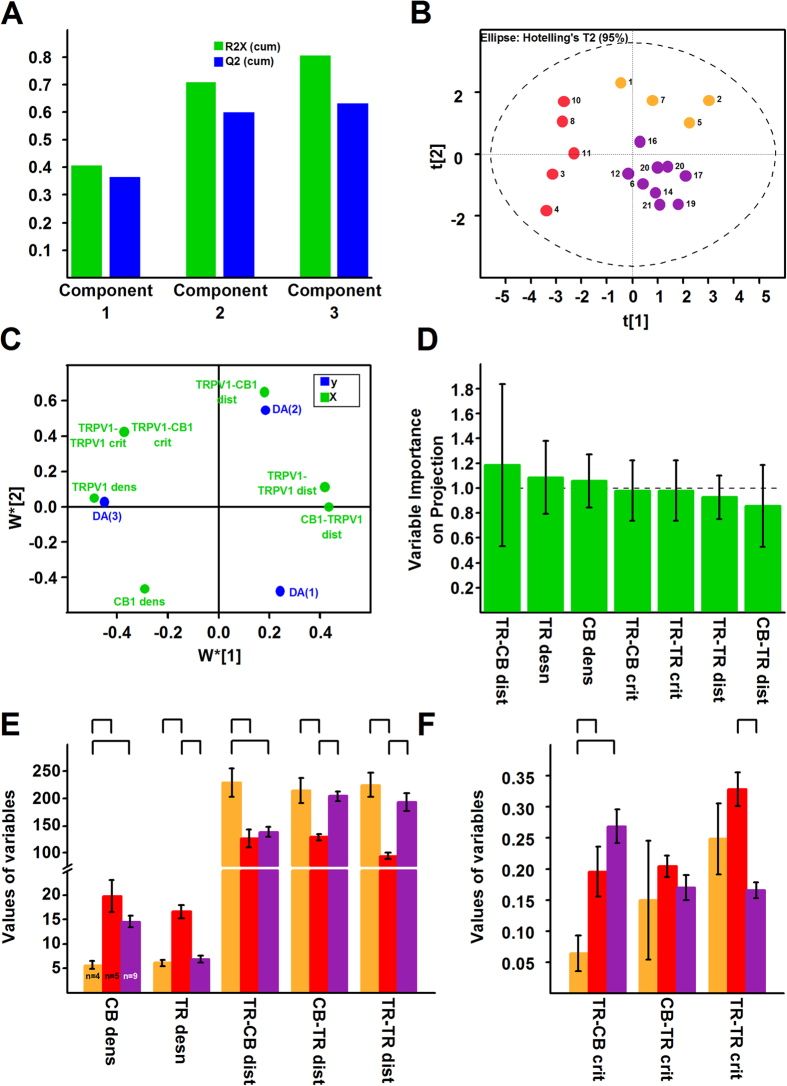
Morphometric analysis of electron microscopic images reveals three types of neurons. (**A**) Based on results of the multivariate statistical tool principal component analysis (PCA; see Supplementary Figure 11) we built a partial least square discriminant analysis (PLS-DA) model to find classify the groups found by PCA. PLS-DA identified three principal components which together accounted for ~80% of the variability of data. (**B**) The PLS-DA score plot reveals the presence of well-separated three groups of neurons. (**C**) The loading plot shows the contribution of the variables to the separation of the three groups. (**D**) The variable importance on projection plot shows that all variables contributed significantly to the separation of the groups. The contribution of TRPV1-CB1 average distance (TR-CB dist), TRPV1 density (TR dens) and CB1 receptor density (CB dens) is highly significant. TR-CB crit: number of TRPV1-CB receptor critical distances; TR-TR crit: number of TRPV1-TRPV1 critical distances; TR-TR dist: TRPV1-TRPV1 average distance; CB-TR dist: CB1 receptor-TRPV1 average distance. (**E**,**F**) Bar charts showing average values of morphometric data of neurons belonging to the three groups shown in (**B**). Statistical analysis (ANOVA followed by Fischer’s post-hoc test) shows that neurons is Group 1 (yellow) are different from neurons both in Group 2 (red) and 3 (purple) in CB1 receptor density (CB dens), TRPV1 – CB1 receptor average distances (TR-CB dist) and the number of TRPV1 – CB1 receptor critical distances (TR-CB crit). Neurons in Group 2 (red) are different from neurons in Group 3 (purple) mainly in the distribution of TRPV1 (TRPV1 density (TR dens); TRPV1-TRPV1 average distance (TR-TR dist) and the number of TRPV-TRPV1 critical distance (TR-TR crit)). CB1 receptor-TRPV1 average distances (CB-TR dist) are also different between neurons in Group 2 and 3. distribution. The number of CB1 receptor TRPV1 critical distances (CB-TR crit) is not different between the 3 groups.

To study the putative groupings in more details, we built a partial least square discriminant analysis (PLS-DA) model, which again suggested the presence of 3 PCs (Fig. 7A). The PLS-DA score plot clearly showed the separation of three major groups (Fig. 7B). The loading plot (Fig. 7C) shows how, while the variable importance of projection plot (Fig. 7D) reveals the significance of, the various morphometric properties contributed to the separation of the membrane patches.

Neurons Membrane patches in Groups 1 (yellow in Fig. 7B) exhibited significantly smaller CB1 receptor densities, larger TRPV1 – CB1 receptor average distances, and fewer TRPV1 – CB1 receptor critical distances than patches in Group 2 (red in Fig. 7B) and Group 3 (purple in Fig. 7B,E,F; Table 2). Patches in Group 2 were different from patches in Group 3 mainly in TRPV1 distribution (Fig. 7E,F; Table 2). Together these data indicate that the distribution of the CB1 receptor and TRPV1 enables an intensive crosstalk in a large proportion of PSN (Group 2 and Group 3 neurons) and, due to the large distances between the two receptors, a limited crosstalk in a small group of neurons (Group 1). Importantly, the proportion of Group 1 – Group 2/Group 3 patches was not significantly different from ACR – COR neuron proportion found in rat or C57BL/6 mice (Table 1).

**Figure 8:**
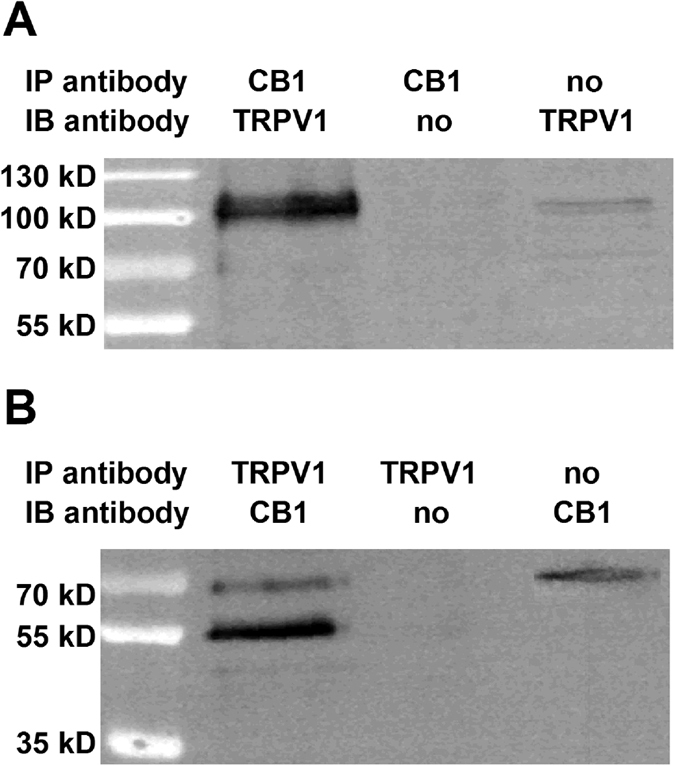
TRPV1 and the CB1 receptor are engaged in PSN. Immunoblots showing TRPV1/CB1 receptor heteromers in the rat DRG. Whole cell lysates were prepared from DRG and immunoprecipitation assays using anti-CB1 (**A**) and anti-TRPV1 (**B**) antibodies were carried out as detailed in Materials and Methods. The resulting immune complexes were analysed by immunoblotting using anti-TRPV1 (**A**) and anti-CB1 (**B**) antibodies. Immunoprecipitating antibodies and immunoblotting antibodies were omitted from positive and negative controls, respectively. TRPV1 was detected as a ~100 kDa band, while the CB1 receptor was detected as a doublet with molecular weight ~55 and ~63 kDa in the immunoprecipitated samples.

The presence of a significant proportion of CB1 receptors and TRPV1 closer than 54.5 nm to each other in PSN suggests that the two receptors, similarly to the cornea^26^, could be engaged in protein-protein interaction. Therefore we next performed immunoprecipitation by either an anti-TRPV1 or an anti-CB1 receptor antibody followed by immunoblotting respectively with an anti-CB1 receptor or anti-TRPV1 antibody on protein samples isolated from rat DRG. We found in both experiments that the immunoprecipiated proteins contained the other molecule (Fig. 8).

## Discussion

We applied a broad range of technologies to address the functional and structural interactions between the CB1 receptor and TRPV1 in PSN. As a major finding, we provide data indicating that spatial distribution of the CB1 receptor and TRPV1 is pivotal for the presence of two types of capsaicin sensitive PSN, the ACR and COR neurons, which are differentiated by their sensitivity to anandamide.

Anandamide, in addition to TRPV1 and the CB1 receptor, also interacts with a series of other molecules^10^,^42^. These interactions could directly or indirectly induce cationic influx into sub-populations of PSN^42^,^43^,^44^. However, anandamide fails to increase the [Ca^2+^]_i_ in PSN of TRPV1^−/−^ mice, and the maximum 30 seconds application time we used is not enough for indirect anandamide-evoked activation^42^. Although we cannot exclude compensatory down-regulation of all anandamide-responsive proteins in TRPV1^−/−^ mice, these findings strongly suggest that the excitatory effect of anandamide depends on TRPV1.

Cultured PSN that respond to capsaicin but not to anandamide have been described previously as a “very small proportion” of neurons^22^. Here we provide unambiguous evidence of pharmacologically distinct COR and ACR phenotypes in capsaicin-sensitive cultured PSN. We demonstrate that COR neurons constitute a significant proportion of cells, which does not depend on the length of culturing (at least between 16 and 74 hours), the order of anandamide or capsaicin application or the concentration of anandamide. However the findings that Biozzi ABH mice, which exhibit a significantly altered endocannabinoid system^30^, possess a significantly lower ratio of COR neurons than Sprague-Dawley rats or CB57BL/6 mice, and that deleting the CB1 receptor in Biozzi ABH mice reduces the proportion of ACR neurons, indicate that the ACR – COR ratio is affected by the endocannabinoid system.

Importantly, CB1 receptor deletion does not reduce the overall proportion of neurons responding to capsaicin. This finding indicates that while capsaicin-sensitivity is an inherent, anandamide-sensitivity is an acquired property of native TRPV1. This behaviour of native TRPV1 is in stark contrast to heterologous TRPV1, which exhibits responsiveness both to capsaicin and anandamide without the CB1 receptor being expressed by the same cell^3^,^45^.

In addition to reducing the proportion of ACR neurons, deleting the CB1 receptor also reduces the amplitude of capsaicin- and anandamide-evoked responses in ACR, but not in COR neurons. Rimonabant, which inhibits both constitutive and ligand-evoked CB1 receptor activation^28^,^29^, also reduces both anandamide- and capsaicin-evoked responses only in ACR neurons. However NESS0327, which blocks ligand-binding to the CB1 receptor^35^, does not reduce responses in either cell types. Together these data, in agreement with recent findings^23^, indicate that the CB1 receptor, through its constitutive activity, produces a sensitising effect on TRPV1 in a significant proportion of PSN.

Considering the sensitising effect of the CB1 receptor on TRPV1, deleting the CB1 receptor could reduce the number of ACR neurons through at least two mechanisms. First, due to the complete loss of the sensitising effect, anandamide-evoked responses could be reduced to below the detection threshold in a major proportion of ACR neurons. Alternatively, due to the loss of the protein-protein interaction, which is indicated by the results of our immunoprecipitation experiment, conformational changes, which could promote anandamide-responsiveness, are lost.

Deleting the CB1 receptor however, does not eliminate anandamide responsiveness. Therefore, mechanisms other than TRPV1 – CB1 crosstalk could also promote anandamide-responsiveness of TRPV1 in PSN. These may include TRPV1 sensitisation/promotion of anandamide-responsiveness by other metabolic receptors, or differing composition of the TRPV1 ion channel. Indeed, while the TRPV1 ion channel is functional either as a homo- or heterotetramer^39^,^40^,^46^,^47^,^48^,^49^, and the SDS-FRL immunolabelling is highly effective in revealing molecules^37^, we found the great majority of TRPV1 molecules to be in isolation in a group of neurons. However, analysis of morphometric data indicate that the number of TRPV1-TRPV1 critical distances might not play a major role in defining ACR or COR phenotype.

Rimonabant increases the time-course of recovery when changes in the [Ca^2+^]_i_ are used to assess responsiveness to capsaicin. However, no similar effect of rimonabant can be observed when responses are assessed by measuring whole-cell currents. Therefore, this rimonabant-evoked effect could be due to the reduced inhibitory effect of the CB1 receptor on voltage-gated Ca^2+^ channels^8^,^9^,^10^ rather than a reduced inhibitory effect on TRPV1.

NESS0327 at 1 nM increases the amplitude of capsaicin-evoked Ca^2+^ transients in ACR neurons, which may also suggest that an inhibitory effect is produced in these cells by CB1 receptor activity evoked, for example, by anandamide synthesised by sub-populations of PSN^31^,^32^,^33^,^27^. However, NESS0327 *per se* increases the [Ca^2+^]_i_ in capsaicin-responding cells. Therefore, in addition to reduced CB1 receptor-mediated inhibition of TRPV1, direct or indirect effects of Ca^2+^ permeable channels or blocking Ca^2+^ sequestration could also underlay this effect.

The lack of a significant CB1 receptor-mediated inhibitory effect on TRPV1 is surprising because a series of reports confirms this effect^16^,^20^,^21^,^22^,^24^,^25^,^26^. However, the anandamide-induced and CB1 receptor-mediated inhibitory effect on TRPV1 is evident at low nM anandamide concentration^16^,^24^,^25^. Therefore, it is reasonable to assume that the downstream effects of anandamide on the CB1 receptor may depend on the concentration of this agent, and only the low nM anandamide-evoked CB1 receptor signalling can overcome the constitutive activity-mediated sensitising effect.

While our single-cell RT-PCR experiments show that all ACR and the majority of COR neurons express the CB1 receptor, the use of rimonabant or the deletion of the CB1 receptor reduces responses only in ACR neurons. Therefore, differing spatial distribution pattern of the two molecules could underlay different responses in the two types of neurons. Morphometric data indeed reveals the presence of three distinct distribution patterns of TRPV1 and CB1 receptors on membrane patches of PSN. Membrane patches belonging to two groups (Group 2 and Group 3; Fig. 7B), which differ from each other mainly in the distribution of TRPV1, express the two receptors in medium/high density and proximity, whereas a smaller group of patches (Group 1; Fig. 7B) expresses the receptors in low density and in isolation. This latter group is differentiated from the rest of the patches by its low CB1 receptor density and the low number or absence of TRPV1-CB1 receptor critical distances. Importantly, our functional studies also suggest that spatial proximity of TRPV1 and the CB1 receptor could be responsible for the anandamide-responsiveness-promoting effects of the CB1 receptor on TRPV1, explaining the presence of ACR and COR neurons. Further, the proportion between ACR and COR neurons and neurons expressing the receptors in high density and close proximity and in low density and isolation are statistically insignificant. Based on these considerations, it is reasonable to hypothesise that neurons expressing the two receptors in high density and close proximity could be associated with the ACR, whereas those expressing the two receptors in low density and in isolation could be COR in phenotype.

In summary, our data demonstrate that the CB1 receptor sensitises TRPV1 and promotes anandamide-responsiveness of TRPV1 in a major sub-population of capsaicin-sensitive PSN, and that spatial proximity of the two molecules may underlay these effects. The sensitising and anandamide-responsiveness-promoting effects in PSN are of particular importance because TRPV1 in these neurons is pivotal in the development of pain in peripheral pathologies^50^,^51^, whereas activation of the CB1 receptor by anandamide in PSN has an analgesic effect^52^,^53^. Therefore, increasing anandamide levels through the inhibition of anandamide hydrolysis by fatty acid amide hydrolase (FAAH) has been suggested as a novel means to control pain associated with peripheral pathologies^53^,^54^,^55^. Our present findings however, indicate that increased tissue levels of anandamide, which seems to have limited TRPV1 desensitising effect^56^, could significantly reduce the potential analgesic efficacy of FAAH inhibitors. Therefore, in order to utilise the analgesic potential of endogenous anandamide fully in PSN, ways to interfere with the mechanisms, through which the CB1 receptor promotes anandamide-responsiveness and sensitisation of TRPV1, must be identified. Due to the co-expression of TRPV1 and the CB1 receptor in various areas of the brain^13^, the CB1 receptor – TRPV1 crosstalk we characterised in the present study could also have relevance in brain signal processing.

## Methods

All procedures were performed in accordance with the UK Animals (Scientific Procedures) Act 1986, the revised National Institutes of Health *Guide for the Care and Use of Laboratory Animals*, the Directive 2010/63/EU of the European Parliament and of the Council on the Protection of Animals Used for Scientific Purposes and the guidelines of the Committee for Research and Ethical Issues of IASP published in Pain, 16 (1983) 109–110. Further, we obeyed to Good Laboratory Practice and ARRIVE guidelines. All procedures on animals were approved by veterinary services (Central Biological Services) at Imperial College London, UK. Every effort was taken to minimize the number of animals used.

### Drugs

The following drugs were used in this study: capsaicin (500 nM or 100 nM, Tocris, UK); anandamide (1 mM to 30 μM, Tocris), rimonabant (200 nM, NIH, USA), mustard oil (50 μM; Sigma), NESS0327 (1nM and 100nM, Cayman Chamicals) and ionomycin (5 μM, Sigma). Anandamide was dissolved in ethanol then the stock solution was prepared with Tocrisolve (Tocris). Capsaicin, rimonabant, mustard oil, NESS0327 and ionomycin stock solutions were prepared in DMSO. The final DMSO dilution was equal or less than 1:2000. The minimum final Tocrisolve and ethanol dilutions were ~1:10^6^ and 1:500, respectively. In control experiments, none of the vehicles at their maximum concentration produced any responses (data not shown).

### Animals

Forty four male Sprague-Dawley rats (80–200 g) and nine wild type (five C57BL/6 ×129SvJ (WT-TRPV1) and 4 Biozzi ABH (WT-CB1) and five TRPV1^−/−^ and four CB1^−/−^ mice (with C57BL/6 ×129SvJ and Biozzi ABH background for TRPV1^−/−^ and CB1^−/−^, respectively) were housed in climate-controlled rooms, on a 12 h light/dark cycle and with food and water *ad libitum*. All mice were male and between 20 and 25 g. Animals were terminally anaesthetised by isoflurane or CO_2_, and decapitated.

### Cell culturing

Dorsal root ganglia from the C1 to the S1 segment were dissected and placed into Ham’s nutrient F12 culture medium (Sigma, UK) supplemented with 1 mM L-glutamine (Invitrogen, UK), 5000 IU/ml penicillin (Invitrogen, UK), 5000 μg/ml streptomycin (Invitrogen, UK) and 2% ultroser G (Biospectra, France). Ganglia were incubated in type IV collagenase (Lorne Diagnostics, UK, 300 U/ml) for 3 hours at 37 °C at 5% CO_2_. Following several washes in the supplemented culture medium, ganglia were triturated with fire-polished Pasteur pipettes, and plated on poly-DL-ornithine (Sigma, UK)-coated glass coverslips in the supplemented medium. Cells were grown in the supplemented medium in the presence of 50 ng/ml nerve growth factor (NGF, Promega, USA) for16–72 hours.

### Electrophysiology

Cells attached to coverslips were placed into a recording chamber and superfused with a bath solution (1–2 ml/min). Borosilicate glass micropipets (4–6 MΩ) were pulled on a DMZ automated electrode puller (DMZ, Germany). Conventional whole-cell voltage-clamp recordings were done from neurons of 15–30 μm in diameter using an Axopatch 200B amplifier, Digidata 1200 digitizer and the pClamp 8 software package (Molecular Devices, USA) with a respective sampling and filtering rate of 1 kHz and 5 kHz at 37 °C. The holding potential was −60 mV.

The bath and pipette solutions contained (concentrations in mM) NaCl 150, KCl 5, MgCl_2_ 2, CaCl_2_ 2, HEPES 10, glucose 10, and NaCl 5, KCl 150, MgCl_2_ 2, HEPES 10, EDTA 1, respectively. The pH of both the bath and pipette solutions was adjusted to pH7.4. Drugs were applied in the bath solution through a small plastic tube (i.d. 0.5 mm) that was placed within ~100 μm from the cell from which the recording was done. Drug application was controlled by the pClamp8 software package, and anandamide and capsaicin were applied two minutes apart. In some experiments neurons were incubated it fluorescein isothiocyanate -conjugated IB4 to identify one of the main sub-populations of nociceptive primary sensory neurons.

### Ca^2+^-imaging

For assessing changes in the [Ca^2+^]_i_, primary sensory neurons cultured for 1–3 days were loaded with Fura-2 acetoxymethyl ester (Fura-2 AM, 5 μM; Molecular Probes, Inc., Eugene, OR) in the presence of 2 mM probenecid (Molecular Probes) for 60 minutes at 37 °C in a HEPES-buffered saline (in mM): NaCl 122; KCl 3.3; CaCl_2_ 1.3; MgSO_4_ 0.4; KH_2_PO_4_ 1.2; HEPES 25; glucose 10; adjusted with NaOH to pH 7.4. Coverslips were placed in a laminar flow perfusion chamber (Warner Instrument Corp., UK) and superfused with extracellular solution (in mM: NaCl 160; KCl 2.5; CaCl_2_ 1; MgCl_2_ 2; HEPES 10; glucose 10; pH 7.4) continuously via a six-channel perfusion system. The following test solutions were applied to the cells: anandamide (30 μM, for 30 seconds), capsaicin (100 nM for 15 seconds, or 500 nM for 30 seconds), mustard oil (50 μM for 30 seconds; Sigma) and KCl (50 mM for 30 seconds). The outlet of the perfusion system was positioned next to the group of neurons from which the recordings were obtained. In some experiments, ionomycin (5 μM, Sigma) was also used as control for maximally increasing the intracellular calcium concentration at the end of the recordings. Application of drugs was controlled manually. Experiments were performed at 37 °C. Only one field of view was tested on each coverslip.

Imaging was performed on either of two systems. Images of Fura-2 loaded cells with the excitation wavelength alternating between 340 or 355 and 380 nM were captured with a Peltier element-cooled slow scan charge-coupled device camera system (PTI, NJ, USA or optiMOS, QImiging, Canada). Following subtraction of the background fluorescence, the ratio of fluorescence intensity at the two wavelengths as a function of time (rate 1 Hz) was calculated automatically (*R* = *F*340/*F*380 or *R* = *F*355/*F*380) either by the ImageMaster 5.0 software package (PTI) or the WinFlour software package (Dr Dempster, Strathclyde University, UK). Data were further analysed by the pClamp 10 software package (Molecular Devices, USA).

### Single-cell polymerase chain reaction

Following whole-cell voltage-clamp recordings, neurons were collected by drawing them into large bore electrodes made from borosilicate glass capillaries. Each cell was then placed in RT-PCR tubes with 3 μl 3.3x RT-PCR buffer, 5 μl nuclease free water and sonicated for 60 s. The 3.3x RT-PCR buffer consisted of 5x First Strand Buffer (66 μl), 10 mM dDNTP Mixture (16.5 μl), 3 μg/μl Random Primers (4 μl) and nuclease free water (13 μl). One μl DTT (Invitrogen, USA), 0.5 μl Superscript II (Invitrogen, USA) and 0.5 μl RNase inhibitor (Promega, USA) were then added to each tube and the RNA was reverse transcribed into cDNA at 37 °C for 2 hours. cDNA produced was then stored at −20 °C.

Each cDNA sample was used for 30 cycles of GoTaq DNA polymerase (Promega)-catalysed PCR with primers designed to amplify the CB1 receptor and GAPDH cDNA. The sequences of the primers were as follows: CB1 receptor forward 5′-TCCATAGAGAAGGGTATTGATAGAAT-3′ and reverse 5′-TTTGTAGCTGTGAGAAGAGGTG-3′ expected size 127 bp, and GAPDH forward 5′-GGTGAAGGTCGGAGTCAACG-3′ and reverse 5′-CAAAGTTGTCATGGATGACC-3′, expected size 380 bp. The PCR reaction mixture contained cDNA, forward and reverse primers, 1 mM MgCl_2_, 1× Green Go-Taq Reaction buffer (Promega), 0.2 mM deoxynucleotide mix (Promega) and 1.25 U Go-Taq DNA polymerase (Promega). Each amplification cycle consisted of denaturation at 95 °C for 30 s, annealing at 57 °C for 60 s. PCR products were then used as a template for a second round reaction for the CB1 receptor using the same primers and conditions. Products were separated on 2% agarose gel by electrophoresis and visualized by ethidium bromide. Signals were analyzed with the Syngene software.

### Combined immunofluorescence staining

Cultured rat PSN attached to the coverslips were washed with PBS and fixed by 4% paraformaldehyde in 0.01 M PBS (pH 7.4) for 10 minutes. Intact lumbar DRGs were collected following transcardial perfusion of terminally anaesthetised rats and mice by 4% paraformaldehyde. DRGs were post-fixed in 4% paraformaldehyde, washed in 0.01 M PBS and cut into 10 mm sections. Blocking with 10% bovine serum albumin was followed by incubating the cells and sections with an anti-TRPV1 (AB5370P, Chemicon, raised in rabbit against 21 amino acids of the C terminus (TGSLKPEDAEVFKDSMVPGEK), diluted at 1:1000) and an anti-CB1 receptor (Prof. K. Mackie, raised in goat against 72 amino acids (SKDLRHAFRSMFPSCEGTAQPLDNSMGDSDCLHKHANNTASMHRAAESCIKSTVKIAKVTMSVSTDTSAEAL) of the C terminus, diluted at 1:8000; Veress *et al*. 2014) antibody overnight, at 4 °C. Staining was visualised by secondary antibodies conjugated with Cyanine 3- and CFTM488A-conjugated secondary antibodies. Coverslips were mounted on glass slides and sections were covered with Vectashield containing DAPI (Vector Laboratories, UK) and examined using an LSM 510 META confocal laser scanning microscope (Zeiss, Oberkochen, Germany). The specificity of the TRPV1 antibody were tested on rat DRG sections (Supplementary Figure 12A) and CB1 receptor knock out mice^57^.

### Sodium dodecyl sulfate (SDS)-digested freeze-fracture replica immunolabeling

Immunohisto-chemical labeling for electron microscopy was performed as described previously^37^. Briefly, adult (150–200 g) male Sprague-Dawley rats (n = 4) were anesthetized with sodium pentobarbital (50 mg/kg, i.p.), and perfused transcardially with 25 mM PBS, followed by 2% paraformaldehyde and 15% saturated picric acid in 0.1 M phosphate buffer (PB). Sections from DRGs were cut at a thickness of 110 μm. The slices were cryoprotected in a solution containing 30% glycerol in 0.1 M PB and then frozen by a high-pressure freezing machine (HPM 100; Leica, Austria). Frozen samples were inserted into a double replica table and fractured into two pieces at −130 °C and replicated by carbon deposition (5 nm thick), platinum (2 nm), and carbon (15 nm) in a freeze-fracture replica machine (BAF 060; BAL-TEC, Lichtenstein). They were digested in a solution containing 2.5% SDS and 20% sucrose in 15 mM Tris-HCl at 80 °C for 18 h then at 60 °C for 18 h. The replicas were washed in 25 mM PBS containing 0.05% bovine serum albumin (BSA; Roth, Germany) and then incubated in a blocking solution containing 5% BSA for 1 h. Subsequently, replicas were incubated in a mixture of primary antibodies (TRPV1 raised in guinea pig against the 22 amino acids of the C terminus (YTGSLKPEDAEVFKDSMVPGEK; Neuromics, USA), the dilution was 1:1000 and CB1 receptor (Prof. K. Mackie, raised in goat; Veress *et al*. 2014), dilution was 1:4000) in 50 mM Tris-buffered saline (TBS) containing 1% BSA. After several washes, replicas were reacted with a mixture of gold-coupled donkey anti-guinea pig (for TRPV1; 12 nm) and rabbit anti-goat (for CB1, 5 nm) secondary antibodies (1:30; BioCell Research Laboratories, Cardiff, UK) in 25 mM TBS containing 5% BSA. The anti-TRPV1 antibody from Neuromics provided better staining in the replicas than then the anti-TRPV1 antibody from Chemicon, which we tried in a pilot study. They were then washed and picked up on 100-mesh grids. The specificity of the antibodies was tested on tissues dissected from TRPV1 and CB1 knock out mice^57^,^58^.

### Immunoprecipitation

Rat DRGs were homogenized in ice-cold NP40 buffer (Thermo Fisher Scientific, Rockford, IL) supplemented with 1 mM PMSF (phenylmethylsulfonyl fluoride; Sigma-Aldrich, St. Louis, MO) and protease inhibitor cocktail (Sigma-Aldrich) in a HT mini homogenizer (OPS Diagnostics, Lebanon, NJ) using zirconium beads (OPS Diagnostics). Homogenates were sonicated twice for 10 sec each and agitated constantly for 2 hours at 4 °C. Lysates were centrifuged for 20 minutes at 12,000 rpm at 4 °C. The protein concentration of the supernatant was determined by BCA assay (Thermo Fisher Scientific).

Immunoprecipitation of 350–500 μg cell lysate was carried out overnight with 2 μg rabbit anti-CB1 (H-150; Santa Cruz Biotechnology, Heidelberg, Germany) that was raised against the 150 amino acids of the C terminus of the human CB1 receptor (MKSILDGLADTTFRTITTDLLYVGSNDIQYEDIKGDMASKLGYFPQKFPLTSFRGSPFQEKMTAGDNPQLVPADQVNITEFYNKSLSSFKENEENIQCGENFMDIECFMVLNPSQQLAIAVLSLTLGTFTVLENLLVLCVILHSRSLRC). The specificity of this antibody was studied by Western blotting on brain tissues isolated from CB1 knock out mice (Supplementary Figure 12B). In another experiment, the immunoprecipitation was done by a goat anti-TRPV1 (P-19; Santa Cruz Biotechnology) antibody. The specificity of this antibody was shown previously^59^. In addition we also studied the specificity by Western blotting (Supplementary Figure 12C). Samples were constantly agitated at 4 °C. Immunoprecipitating antibodies were omitted from negative controls (mock control). Antibody/antigen complexes were then precipitated with 20 μl protein A/G PLUS agarose (Santa Cruz Biotechnology) for 2 h at 4 °C. Agarose/antibody complexes were collected by centrifugation at 2500 rpm for 5 minutes and the pellet was washed four times in ice-cold PBS supplemented with 1 mM PMSF and protease inhibitor cocktail. The bound antigen was eluted in 2x concentrate dithiothreitol-containing sample buffer by heating to 50 °C for 10 min. The anti-TRPV1 and anti-CB1 receptor antibodies we used for immunoprecipitation provided better signal to noise ration than the antibodies used for immunolabelling.

Immunoprecipitated samples and whole cell lysates (40–60 μg protein; input) were analysed on 10% SDS polyacrylamide gel under reducing conditions. Immunoblotting was carried out using goat anti-TRPV1 (P-19; Santa Cruz Biotechnology; 1:750) or goat anti-CB1 (K-15; Santa Cruz Biotechnology; 1:1000) primary antibodies. Appropriate secondary antibodies were purchased from Santa Cruz Biotechnology. Immunoreactive bands were visualised by the SuperSignal West Pico Chemiluminescent Substrate (Thermo Fisher Scientific) using a Gel Logic 1500 Imaging System (Kodak, Tokyo, Japan). The specificity of the K-15 anti-CB1 receptor antibody was assessed by Western blotting using tissues from WT and CB1^−/−^ mice (Supplementary Figure 12D).

### Data analysis and statistics

Whole-cell recordings were analysed with the ClampFit 8.0 software package. Cells were regarded as anandamide- or capsaicin-responsive if the drug application-associated change in the current exceeded 50 pA.

Calcium imaging data were analysed by a custom-made Microsoft Excel macro, which following visual inspection of the time-Δratio (340/380 or 355/380) line chart, automatically measures the peak amplitude of the calcium transients produced by each drug. An increase of more than 10% in the 340/380 (or 355/380) ratio, if it was visibly associated with drug application, was regarded as a response. Based on previous data^27^, 2.5 times of the standard deviation of the amplitude of the baseline noise equals with a maximum of 9.5–10% increase in the 340/380 (or 355/380) ratio.

Comparison of KCl-evoked increase in the [Ca^2+^]i in capsaicin-sensitive neurons isolated from WT-CB1 and CB1^−/−^ mice revealed that deletion of the CB1 receptor significantly reduces the depolarisation-induced changes in the [Ca^2+^]_i_ (from 0.73 ± 0.04 (n = 122) to 0.48 ± 0.02 (n = 112), p < 0.0001, Student’s t-test). Therefore, we compared the absolute values of anandamide- and capsaicin-induced changes in [Ca^2+^]_i_ in all calcium imaging experiments.

Electron microscopic morphometric data were analysed by PCA followed by PLS-DA. PCA is an unsupervised multivariate statistical tool which transforms a large number of variables into a smaller number of uncorrelated variables known as PCs. PCA generates clusters the separation of which is enhanced by PLS-DA. PLS-DA itself is a supervised classification tool which rotates PCA component to achieve the largest separation. PLS-DA also provides the information on the variables which contribute the most to the separation.

The normality of data was checked by the Kolgomorov-Smirnov test. Data obtained by the same drug application were averaged. The effects of various treatments were compared with paired or unpaired one-tailed or two-tailed Student’s t-test, analysis of variances (ANOVA) followed by Fisher’s post-hoc test, or by two-sided Fisher’s exact test as appropriate. Differences were regarded significant at p < 0.05. In Results, the exact p values are given unless it is less than 0.0001. In this latter case, p values are indicated as p < 0.0001. Data are expressed as mean ± standard error of mean; “n” refers to the number of cells or cultures used in the same measurements as indicated.

## Additional Information

**How to cite this article**: Chen, J. *et al*. Spatial Distribution of the Cannabinoid Type 1 and Capsaicin Receptors May Contribute to the Complexity of Their Crosstalk. *Sci. Rep.* **6**, 33307; doi: 10.1038/srep33307 (2016).

## Supplementary Material

Supplementary Information

## Figures and Tables

**Table 1:** Number of cells found in different categories in various experiments. Significance values refer to the results of Fischer’s exact p value. Bold indicates significant difference.

Electrophysiology/Ca2+ imaging					
Number of experiment	Description of experiment	Total number of capsaicin-responding neurons	Number of ACR neurons	Number of COR neurons	Significant difference
1	Whole-cell recordings from rat cultured PSN in control bath solution	39	29	10	From No 2: p = 0.82 From No 3: p = 0.65 From No 5: p = 0.1 From No 6: p = 0.36 **From No 7: p = 0.009** From No 9: p = 0.28 From No 10: p = 0.4 From No 11: p = 1 From No 12: p = 0.08 From No 15: p = 1
2	Ca2+ imaging on rat PSN cultures	57	40	17	From No 1: p = 0.82 From No 3: p = 1 From No 9: p = 0.07 From No 10: p = 0.11
3	Ca2+ imaging in WT-TRPV1 mouse PSN cultures	54	37	17	From No 1 p = 0.65 From No 2: p = 1
4	Ca2+ imaging in TRPV1^−/−^ mouse PSN cultures	0	n/a	n/a	n/a
5	Whole-cell recordings from IB4+ and IB4- cells	25	13	12	From No 1: p = 0.1 From No 6: 0.76 From No 12: p = 0.8
6	Whole-cell recordings from rat cultured PSN in the presence of rimonabant	18	11	7	From No 1: p = 0.36 From No 5: p = 0.76 From No 12: p = 0.79
7	Ca2+ imaging in WT-CB1 mouse PSN cultures	124	113	11	**From No 1: p = 0.009 From No 2: p = 0.0005 From No 3: p = 0.016**
8	Ca2+ imaging in CB1^−/−^ mouse PSN cultures	115	74	41	**From No 7: p < 0.0001**
9	Ca2+ imaging in rat PSN cultures with *a priori* capsaicin application	197	161	36	From No 1: p = 0.28 From No 2: p = 0.07 From No 10: p = 0.71
10	Ca2+ imaging in rat PSN cultures with *a priori* capsaicin application and rimonabant application	237	190	47	From No 1: p = 0.4 From No 2: p = 0.11 From No 9: p = 0.71
11	Ca2+ imaging in rat PSN cultures with *a priori* capsaicin application and 1 μM NESS0237 application	34	25	9	From No 1: p = 1 From No 2: p = 0.81 From No 9: p = 0.35
12	Whole-cell recordings from rat cultured PSN for single cell PCR	56	31	25	From No 1: p = 0.08 From No 5: p = 0.8 From No 12: p = 0.79
**Single-cell polymerase chain reaction**					
Number of experiment	Description of experiment	CB1 receptor expression in ACR neurons	CB1 receptor expression in COR neurons	CB1 receptor expression in NR neurons	Significant difference
13	Single-cell PCR using RNA from rat cultured PSN	11/11	8/10	4/8	n/a
**Immunofluorescent staining**					
Number of experiment	Description of experiment	Total number of TRPV1-expressing cells	Number of TRPV1 and CB1 receptor expressing cells	Significant difference	
14	Immunofluorescent staining	150	149	From number of capsaicin-responsive/CB1 mRNA expressing cells in No 12: p = 0.87	
**Electron microcopy**					
Number of experiment	Description of experiment	Total number of membrane patches analysed	Number of patches with high/medium receptor density	Number of patches with low receptor density	Significant difference from ACR/COR ratio
15	Immuno- electron microcopy	18	14	4	From No 1: p = 1 From No 2: p = 0.76 From No 9: p = 0.75 From No 12: p = 0.1

**Table 2:** Morphometric data of primary sensory neurons examined with SSD-FRL electron microscopy.

No of membrane patch	Number of CB1 receptors	Number of TRPV1	Area of membrane patch (μm^2^)	TRPV1-CB1 average distance (nm)	CB1-TRPV1 average distance (nm)	TRPV1-TRPV1 average distance (nm)	Number of TRPV1-CB1 critical distances	Number of CB1-TRPV1 critical distances	Number of TRPV1-TRPV1 critical distances
1	10	14	1.8	202.9	152.7	199.9	0	0	5
2	5	8	1.3	305.5	215	276	1	1	1
3	152	97	6.2	95.7	127.4	85.7	24	29	32
4	121	83	4	83.8	125.1	80.9	25	25	21
5	27	28	4.8	222.8	231.8	247.2	1	0	5
6	96	27	5.6	130.2	169.6	174.9	7	26	5
7	35	21	4.6	189.5	259.8	179.8	2	14	7
8	62	83	4.5	164	109.7	116	6	14	28
9	16	16	5.9	317.6	399.4	308.4	0	0	3
10	40	38	3	160.7	141.4	93.1	8	10	16
11	116	109	6.9	126.3	142.5	95.5	16	17	33
12	74	64	5.6	146	172.5	126.9	10	8	11
13	357	104	6.4	57.1	93.6	126.8	57	108	19
14	85	52	5	138.5	230	177.5	15	10	7
15	79	21	4.5	82.6	159.6	340.1	8	16	0
16	67	47	7.8	162.6	185.2	135.3	15	15	11
17	85	41	7.5	172.4	192.9	243.7	5	13	4
18	95	52	7.9	152.1	206.9	176.4	16	18	9
19	75	21	3.9	105	238.2	273.2	8	16	3
20	77	33	5.3	154.1	220.4	235.7	10	12	6
21	119	36	1.8	87	228.6	204	10	11	6
