# Supplementary material for: Spatial Distribution of the Cannabinoid Type 1 and Capsaicin Receptors May Contribute to the Complexity of Their Crosstalk
Source: Sci Rep. 2016 Sep 22;6:33307. doi: 10.1038/srep33307 (PMC5032030; doi:10.1038/srep33307)
Supplement: Supplementary Information [file srep33307-s1.doc]

**SUPPLEMENTARY INFORMATION**

**SPATIAL DISTRIBUTION OF THE CANNABINOID TYPE 1 AND CAPSAICIN RECEPTORS MAY CONTRIBUTE TO THE COMPLEXITY OF THEIR CROSSTALK**

Jie Chen*^1, 2^, Angelika Varga*^1,3^, Srikumaran Selvarajah^1^, Agnes Jenes^3^, Beatrix Dienes^3^, Joao Sousa-Valente^1^, Akos Kulik^4,5^; Gabor Veress^6^, Sue Brain^7^, David Baker^8^, Laszlo Urban^9^, Ken Mackie^10^ and Istvan Nagy^1$^

^1^Section of Anaesthetics, Pain Medicine and Intensive Care, Department of Surgery and Cancer, Faculty of Medicine, Imperial College London, Chelsea and Westminster Hospital, 369 Fulham Road, London, SW10 9NH, UK; ^2^Department of Anaesthesiology, Southwest Hospital, Third Military Medical University, Gaotanyan 19 Street, Shapingba, Chongqing 400038, P. R. China; ^3^Department of Physiology, Faculty of Medicine, University of Debrecen, Nagyerdei krt. 98, Debrecen, H-4012, Hungary; ^4^Institute of Physiology, University of Freiburg, Germany D-79104 Freiburg, Germany; ^5^BIOSS Centre for Biological Signalling Studies, University of Freiburg, D-79104; ^6^Department of Laboratory Medicine, Faculty of Medicine and Health, Örebro University, Örebro, Sweden; ^7^BHF Cardiovascular Centre of Excellence and Centre of Integrative Biomedicine, Cardiovascular Division, King's College London, London SE1 9NH, UK; ^8^Centre for Neuroscience and Trauma, Blizard Institute, Barts and the London School of Medicine and Dentistry, Queen Mary University of London, 4 Newark Street, London, E1 2AT, UK; ^9^Preclinical Secondary Pharmacology, Preclinical Safety, Novartis Institutes for Biommedical Research, Cambridge, MA 01932, USA; ^10^Department of Psychological and Brain Sciences and Program in Neuroscience, Indiana University, The Gill Center, 702 N. Walnut Grove Avenue, Bloomington, IN 47405, USA

**Supplementary Table 1**

**Amplitude of anandamide-evoked whole-cell currents in cultured rat primary sensory neurons in the absence and presence of rimonabant.**

| Concentration | Anandamide-evoked responses used to establish the concentration-response relationship | | Anandamide-evoked responses in ACR neurons without rimonabant | | Anandamide-evoked responses in ACR neurons with rimonabant | |
| --- | --- | --- | --- | --- | --- | --- |
|  | n | Amplitude (nA) | n- | Amplitude (nA) | n | Amplitude (nA) |
| 1µM | 11 | -0.31±0.08 | 5 | -0.22±0.02 |  |  |
| 3µM | 15 | -0.86±0.2 | 4 | -0.81±0.37 |  |  |
| 10µM | 26 | -0.94±0.12 | 13 | -0.88±0.19 |  |  |
| 30µM | 14 | -1.29±0.21 | 7 | -1.07±0.33 | 11 | -0.59±0.18* |

* difference between 30μM anandamide-evoked responses in ACR neurons without rimonabant and 30μM anandamide-evoked responses in ACR neurons with rimonabant, p=0.006 (Student’s t-test)

**Supplementary Table 2**

**Number of ACR and COR neurons in the absence of rimonabant.**

| Concentration | ACR^†^  (number/total number of neurons) | COR^†^  (number/total number of neurons) |
| --- | --- | --- |
| 1µM | 5/6 | 1/6 |
| 3µM | 4/5 | 1/5 |
| 10µM | 13/17 | 4/17 |
| 30µM | 7/11 | 4/11 |

^†^Effect of anandamide concentration in the absence of rimonabant:

p= between 1 and 0.6; Fischer’s exact test

**Supplementary Table 3**

Summary data of recordings from IB4+ and IB4- cultured rat primary sensory neurons

|  | ACR | | | COR | |
| --- | --- | --- | --- | --- | --- |
|  | Number of neurons | Peak amplitude of anandamide-evoked responses (nA) | Peak amplitude of capsaicin-evoked responses (nA) | Number of neurons | Peak amplitude of capsaicin-evoked responses (nA) |
| IB4+ | 6 | -0.56±0.12^$^ | -3.77±0.76*, *** | 6 | -2.11±0.50*, **** |
| IB4- | 7 | -0.33±0.08^$^ | -2.55±0.28**, *** | 6 | -0.99±0.24**, **** |

Statistical difference between the overall ACR – COR ratio between this experiments (n=13 and n=12) and experiment No1 (Table 2): p=0.1 Fisher’s exact test

^$^p=0.14

*p=0.09 (overall capsaicin responses in ACR and COR neurons, p=0.06)

**p=0.002 (overall capsaicin responses in ACR and COR neurons, p=0.06)

***p=0.14

****p=0.07

**Supplementary Figure 1**


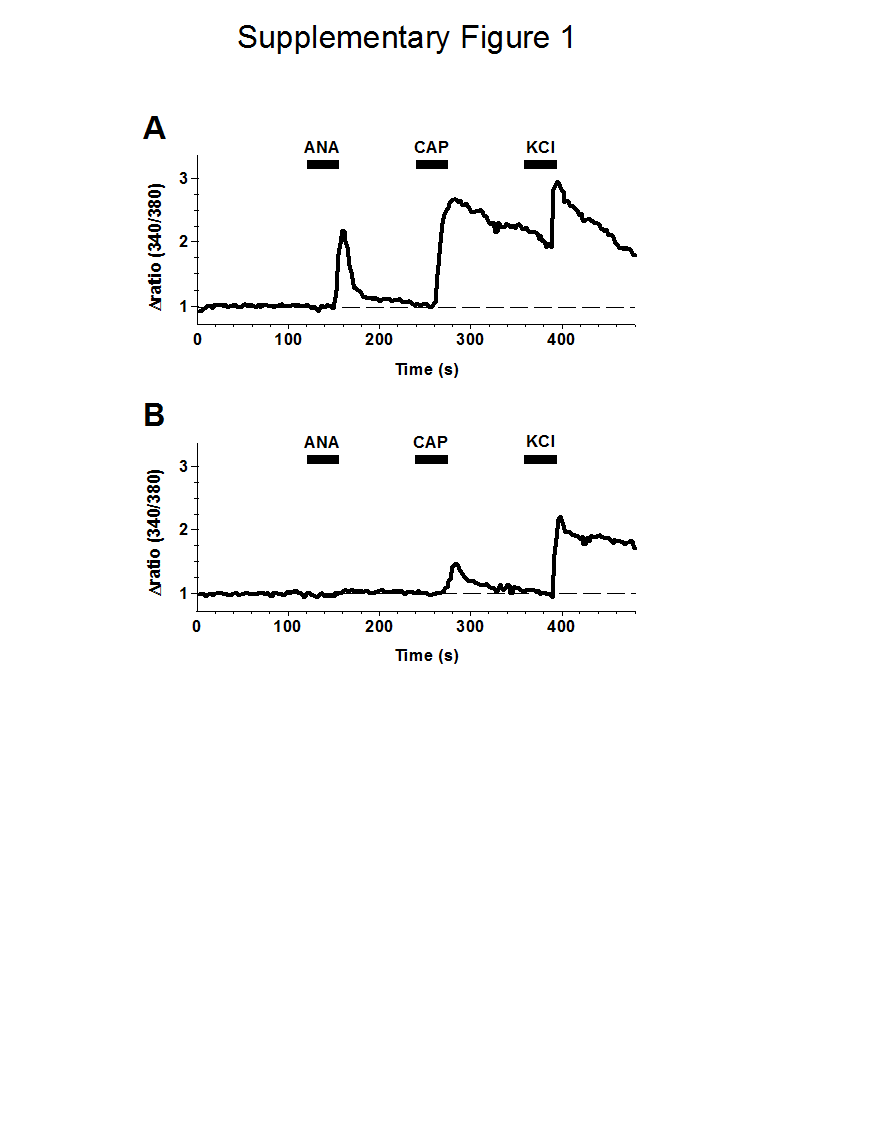


Conventional ratiometric approach was used to provide evidence for the presence of ACR and COR neurons in rat PSN cultures. Cells grew in the culture medium for 16 to 22 hours before loading them with the Ca^2+^ dye fura-2. Neurons were challenged with 30µM anandamide and 500nM capsaicin.

(A) A typical ACR type response. ACR neurons respond to both anandamide and capsaicin.

(B) A typical COR type response. COR neurons respond only to capsaicin. Note also that capsaicin induces smaller responses in COR than in ACR neurons. The ACR – COR neuron ratio we found in this experiment is not different from that found by whole-cell voltage-clamp recordings from rat cultured PSN grown for up to 72 hours.

**Supplementary Figure 2**


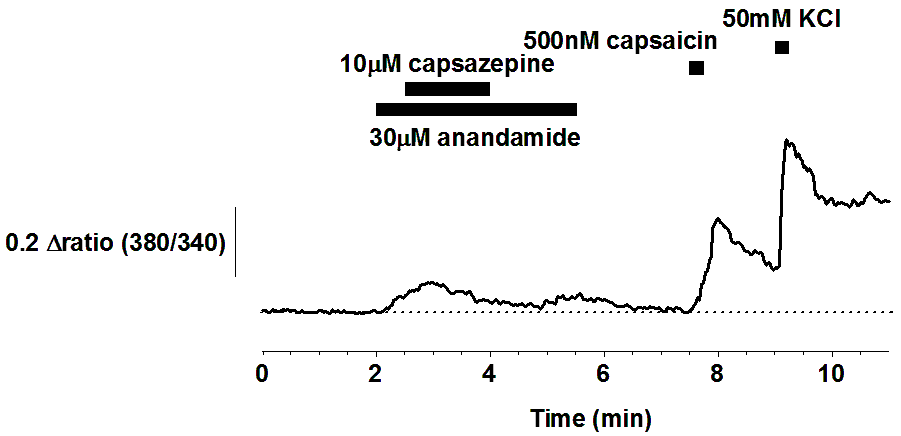


Conventional ratiometric approach was used on rat cultured PSN to provide evidence that the excitatory effect of anandamide is mediated by TRPV1. 30μM anandamide was applied for 30 second then the superfusate was switched to 30μM anandnamide and 10μM capsazepine, a TRPV1 antagonists. After 90 seconds application of 30μM anandnamide and 10μM capsazepine together, the superfusate was switched back to 30μM anandamide alone. Subsequently, capsaicin (500nM) and KCl (50mM) were applied. Anandamide-evoked excitatory effects were invariably blocked by capsaicin.

**Supplementary Figure 3**


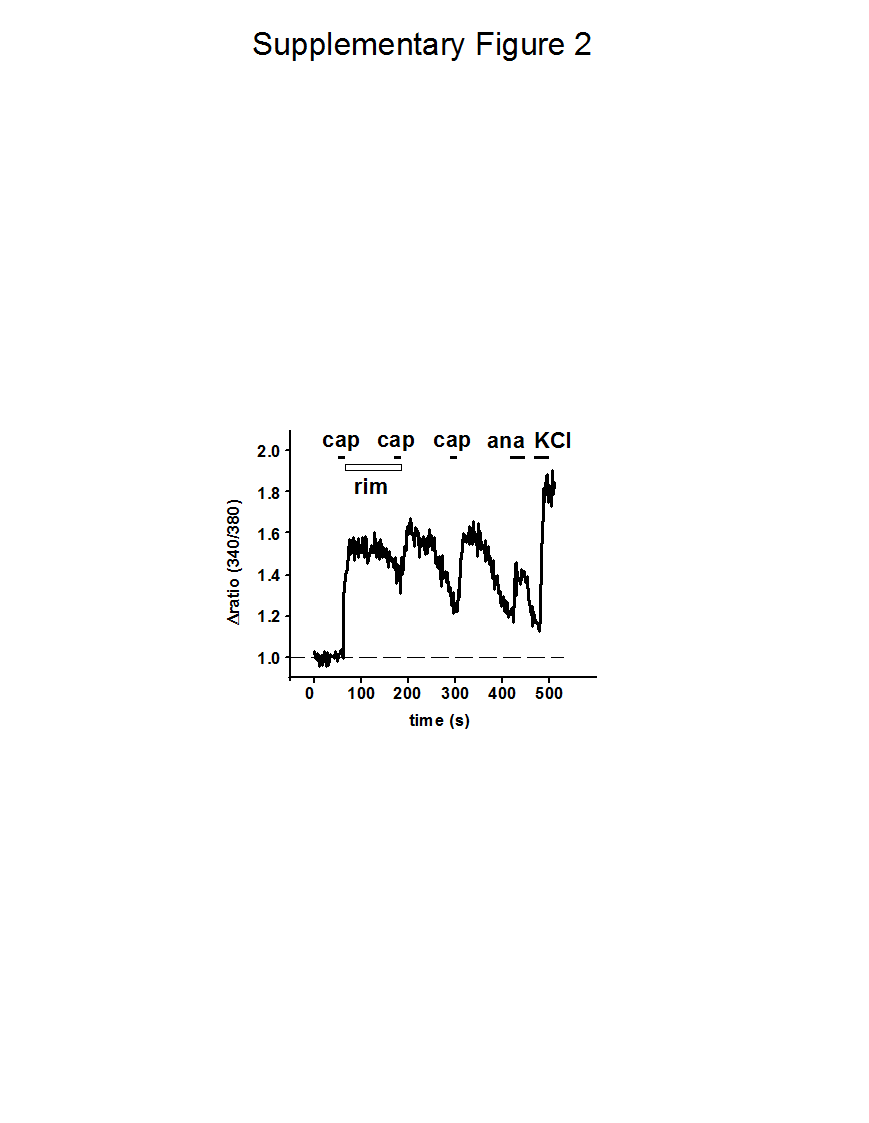


Conventional ratiometric approach was used to assess the effect of 200nM rimonabant (rim) on capsaicin (cap)-evoked responses in rat cultured PSN. Cells were challenged by 100nM capsaicin three times consecutively in every 2 minutes followed by the application of 30μM anandamide (ana) and 50mM KCl. Rimonabant was applied immediately after stopping the first capsaicin challenged until the end of the second capsaicin application. In this ACR type neuron, similarly to a few other ACR neurons and some COR cells, rimonabant appeared to delay recovery.

**Supplementary Figure 4**


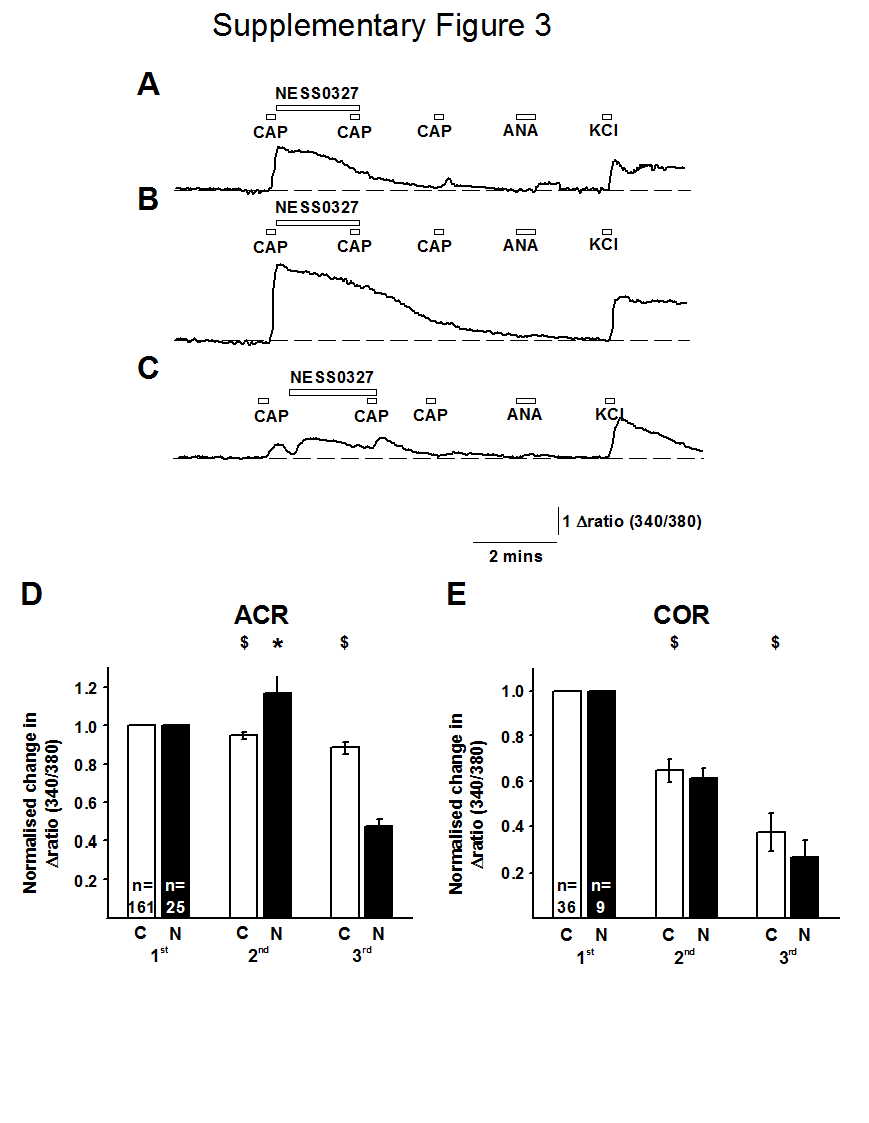


(A)-(C) Conventional ratiometric approach was used to assess the effect of the neutral CB1 receptor antagonist NESS0327 on capsaicin (CAP)-evoked responses in rat cultured PSN. Cells were challenged by 100nM capsaicin three times consecutively in every 2 minutes followed by the application of 30μM anandamide (ANA) and 50mM KCl. NESS0327 (100nM (A) and (B) or 1nM (C)) was applied immediately after the stopping the first capsaicin challenged until the end of the second capsaicin application. At 100nM NESS0327 (A) and (B) invariably delayed the recovery of capsaicin-evoked responses. At 1nM (C) this excitatory effect was less pronounced. When NESS0327 application was delayed by 30 seconds, the effect appeared to be independent of capsaicin application.

(D) Average responses (normalised to the first capsaicin-evoked response) of ACR type neurons in control (C; empty bars) and in the presence of 1nM NESS0327 (N; solid bars). 1nM NESS0327 significantly (*) increased the amplitude of the second (2^nd^) capsaicin-evoked responses. ^$^ indicates significant difference in amplitudes of the second (2^nd^) and third (3^rd^) capsaicin-evoked responses from the amplitude of the first (1^st^) capsaicin-evoked response.

(E) Average responses (normalised to the first capsaicin-evoked response) of COR type neurons in control (C; empty bars) and in the presence of 1nM NESS0327 (N; solid bars). 1nM NESS0327 did not have any effect on the amplitude of the second (2^nd^) capsaicin-evoked responses. ^$^ indicates significant difference in amplitudes of the second (2^nd^) and third (3^rd^) capsaicin-evoked responses from the amplitude of the first (1^st^) capsaicin-evoked response.

**Supplementary Figure 5**


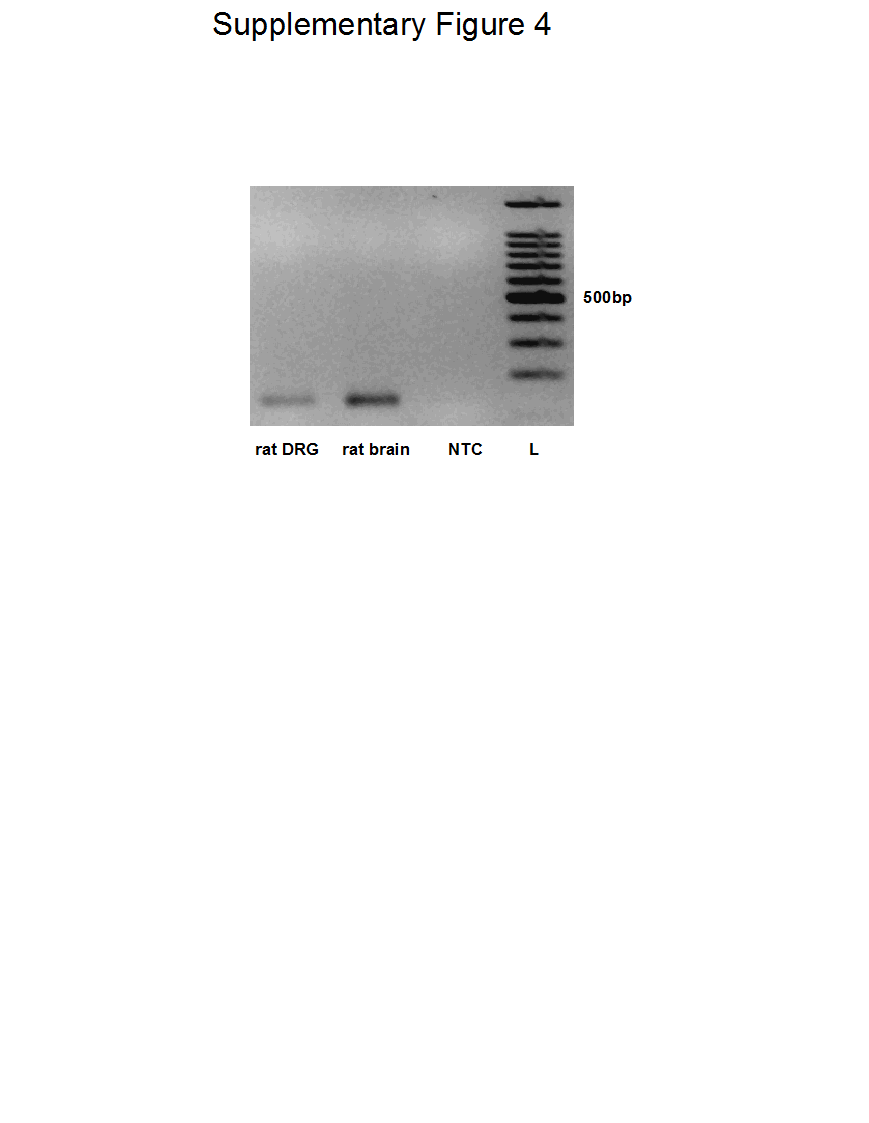


Gel image from a control RT-PCR experiment using cDNA synthesised to RNA isolated from rat DRG and rat brain, or no template (NTC). The CB1 receptor primer produced amplicons (~127kD) which are indistinguishable from the predicted size of the PCR product when cDNA was added to the PCR mixture. However, no amplicon was produced when no template was added to the PCR mixture (NTC). L indicates size marker.

**Supplementary Figure 6**

Confocal microscopic images of cultured primary sensory neurons immunolabelled with anti-TRPV1 (red) and anti-CB1 receptor (green) antibodies. While some neurons expressed the CB1 receptor with low intensity (A2) others exhibited CB1 receptor expression in high intensity at the cytoplasmic membrane (B2). Images of both cells are composed of 6 optical sections of 1μm each. Scale: 20μm

**Supplementary Figure 7**

Confocal microscopic images of a section cut from a rat L4 DRG and immunolabelled with anti-TRPV1 (green) and anti-CB1 receptor (red) antibodies. Blue indicates DAPI staining. Asterisks indicate TRPV1-immunolabelled cells in (A) and TRPV1/CB1 receptor-immunolabelled neurons in (B) which have clearly visible nucleus in the section. In (B), - indicates neurons which exhibit TRPV1- but not CB1 receptor immunolabelling. The great majority of TRPV1-immunolabelled cells also exhibit immunolabelling for the CB1 receptor. Images are composed of 5 optical sections of 0.9μm each. Scale: 50μm

**Supplementary Figure 8**

Images of a single optical section of 0.9 μm of a section cut from a rat L4 DRG and immunolabelled with anti-TRPV1 (green) and anti-CB1 receptor (red) antibodies. Blue indicates DAPI staining. Neither the TRPV1 nor the CB1 receptor immunolabelling exhibits any obvious heterogeneity in their distribution on the cytoplasmic membrane. Scale: 20μm

**Supplementary Figure 9**


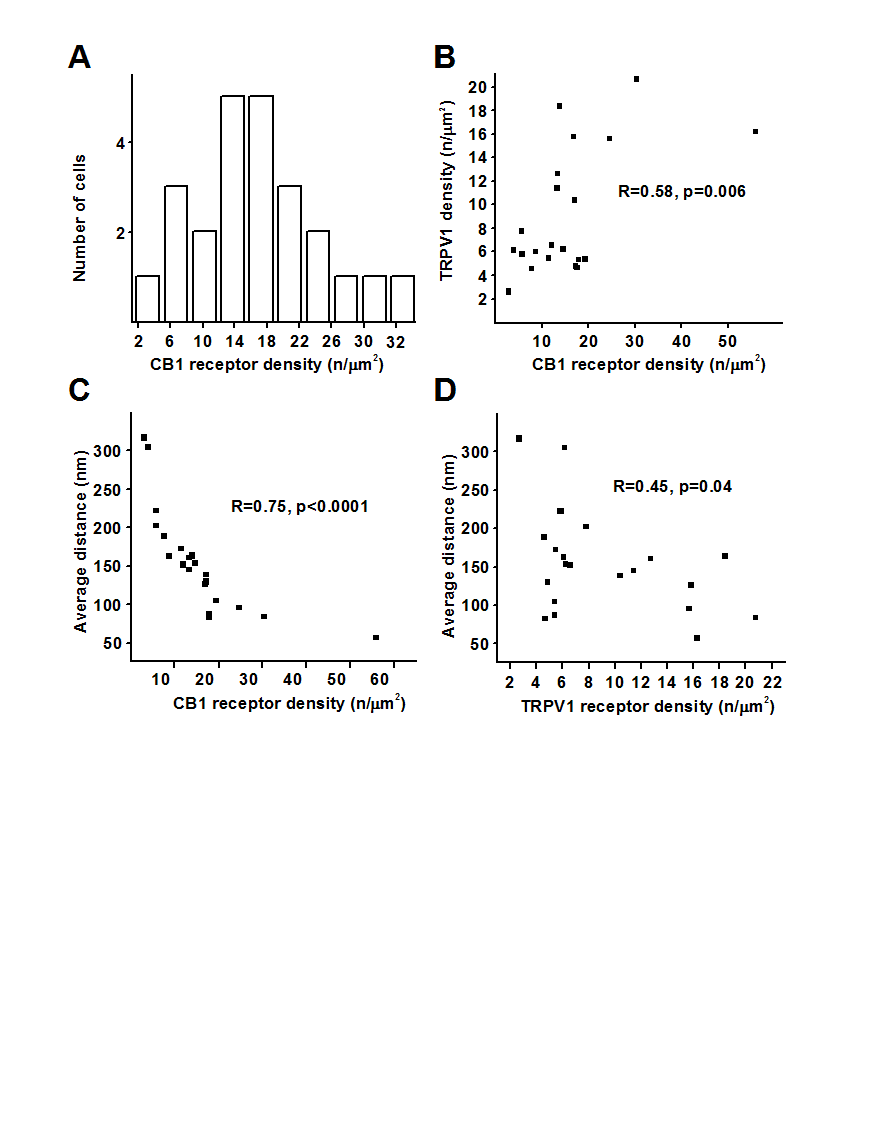


(A) Frequency distribution of CB1 receptor density established on 21 membrane patches of PSN. Based on the CB1 receptor density more than one sub-population of cells exists.

(B) CB1 receptor and TRPV1 receptor densities show a modest correlation.

(C) CB1 receptor density and the average distance between each TRPV1 and the closest CB1 receptor neighbour shows a strong correlation.

(D) TRPV1 density and the average distance between each TRPV1 and the closest CB1 receptor neighbour shows a weak correlation.

**Supplementary Figure 10**


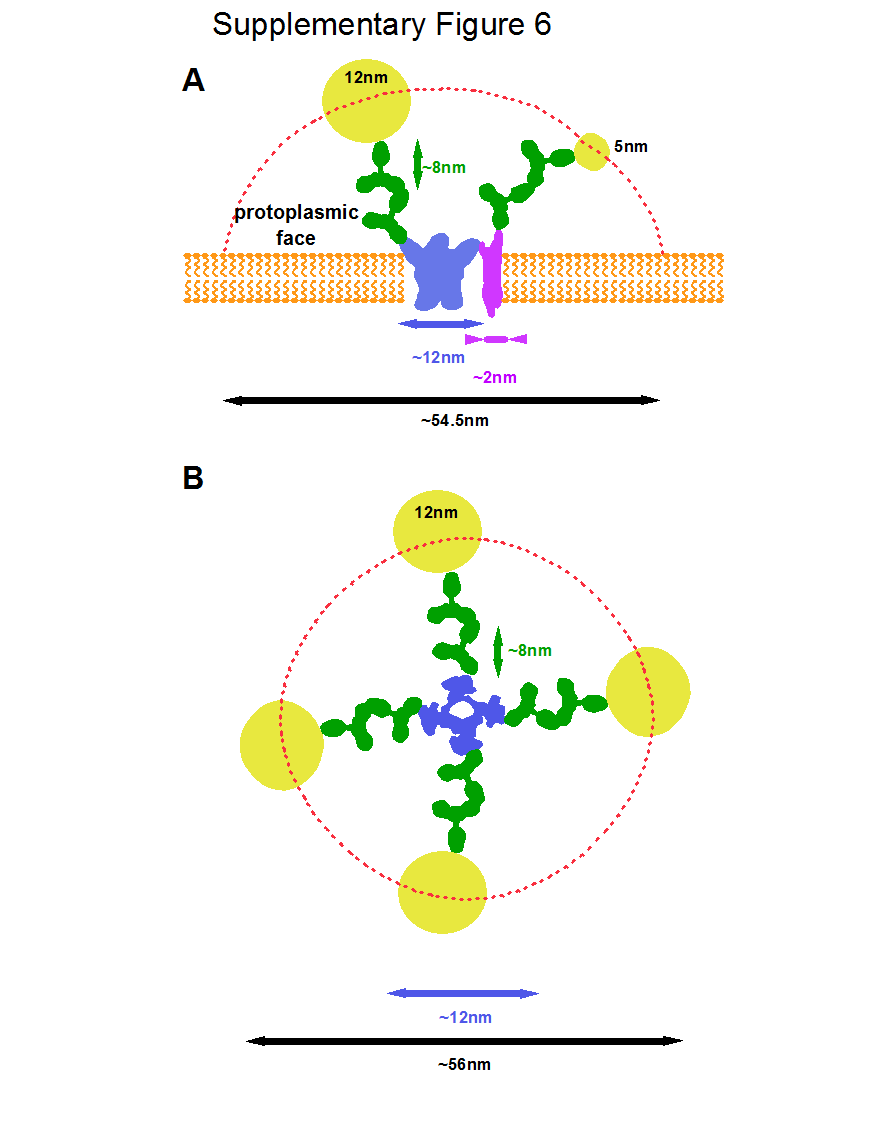


Consideration of calculating distances between gold particles indicating the expression of the CB1 receptor and TRPV1 if the molecules are next to each other.

(A) If the distance between the centre gold particles of different sizes is less than ~54.5nM, the CB1 receptor and TRPV1 are highly likely to be expressed next to each other.

(B) If the distance between the centre gold particles indicating TRPV1 expression is less than ~56nM, TRPV1 molecules are highly likely to form polymers.

**Supplementary Figure 11**


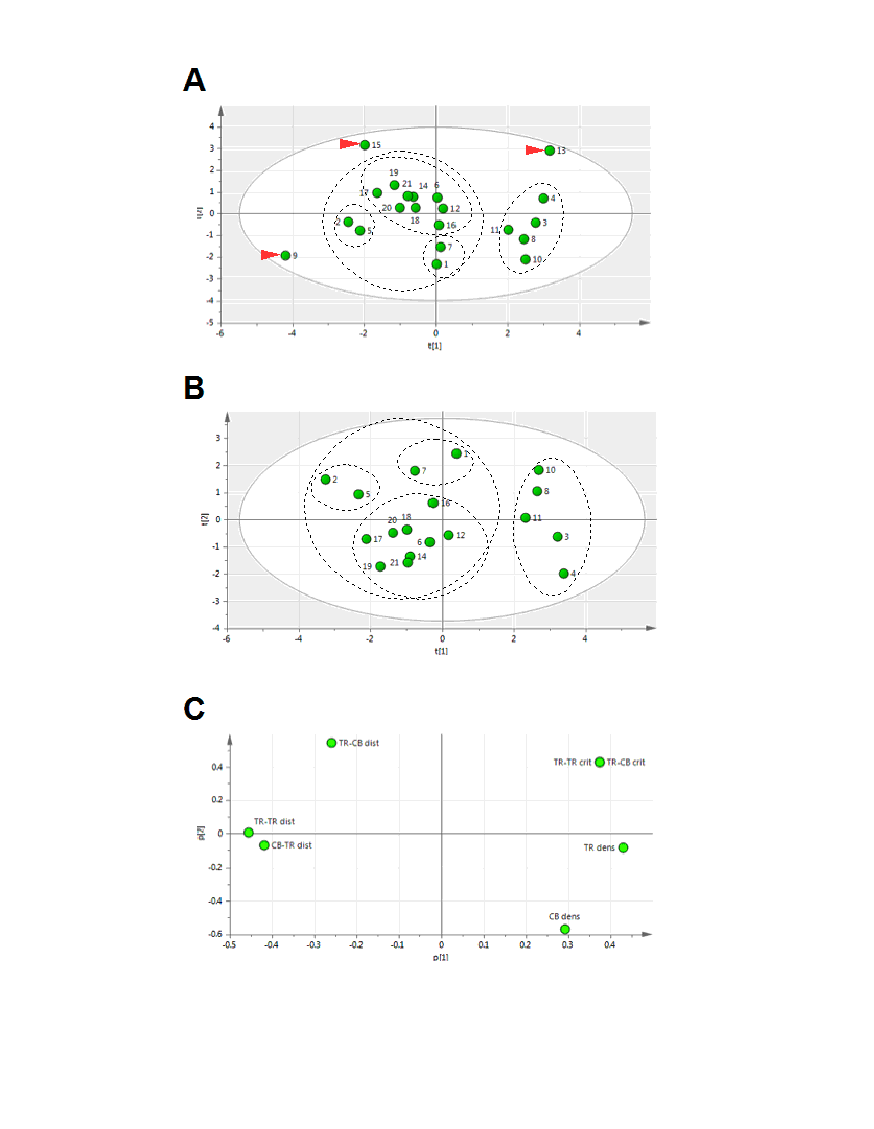


(A) PCA scoring plot when all the variables of all the 21 cells are used to find whether the variables define any grouping. The plot shows the possibility of the presence of 2-4 groups shown by the dotted ellipses. The plot also shows 3 outliers shown by the red arrowheads.

(B) PCA scoring plot following the removal of the three outliers and the variable “number of CB1 receptor – TRPV1 critical distances” which is shown not to contribute significantly to the separation of the groups. The new scoring plot again suggests the presence of 2-4 groups of cells shown by the dotted ellipses.

(C) The PCA loading plot reveals how variables contribute to the separation.

Abbreviations: CB dens: CB1 receptor density, TR-CB dist: TRPV1 – CB1 receptor average distance. TR-CB crit: number of TRPV1 – CB1 receptor critical distances, TR dens: TRPV1 density, TR-TR dist: TRPV1-TRPV1 average distance, TR-TR crit: number of TRPV-TRPV1 critical distances, CB-TR dist: CB1 receptor-TRPV1 average distance.

**Supplementary Figure 12**

(A_1_-A_3_) The anti-TRPV1 antibody (TGSLKPEDAEVFKDSMVPGEK; AB5370P, Chemicon, red) recognises only human and rat TRPV1. Hence, we studied co-immunolabelling of this antibody and the anti-TRPV1 antibody manufactured by Neuromics in guinea pig (GP, green; Baiou et al, 2007) on rat DRG sections. On 8 sections, about 97% of the cells exhibiting immunopositivity for the Neuromics antibody also exhibited immunopositivity with the Chemicon antibody, and 100% of the immunopositive cells with the Chemicon antibody exhibited immunopositivity with the Neuromics antibody. Asterisks indicate neurons which are immunopositive with the Neuromics, but apparently immunonegative with the Chemicon antibody. Scale=50μm.

(B) Protein samples from the brain of wild type (WT) mice and mice lacking the CB1 receptor between the 32 and 488 amino acids (KO) were reacted with the H-150 anti-CB1 receptor antibody, a significant part of the epitope of which might be present in the cells. The anti-CB1 receptor antibody recognises a protein (arrowhead) with the predicted molecular weight (~63kD) only in the WT sample. The arrow with the cross indicates loading control (β-actin).

(C) Samples from the DRG of wild type (WT) and TRPV1^-/-^ (KO) mice were reacted with the P-19 anti-TRPV1 antibody. Samples from WT mice exhibit several proteins; the weak band at around 110kD corresponds to the glycosylated form of TRPV1, the two stronger bands at around 95kD and 88kD (arrow) correspond respectively to the non-glycosylated form of TRPV1 and the TRPV1 splice variant, TRPV1b. The protein at ~60kD could be another TRPV1 splice variant. These proteins are missing in samples from KO mice.

(D) Samples from the brain of a wild type (WT) and a CB1^-/-^ (KO) mouse were reacted with the K-15 anti-CB1 receptor antibody. The anti-CB1 receptor antibody was raised against an epitope near the C-terminus, a part of which might be expressed in the KO mice. The anti-CB1 receptor antibody recognises a protein (arrowhead) the molecular weight of which is similar to that of the CB1 receptor (~63kD) only in the sample from the WT mouse. The arrow with the cross indicates loading control (anti-β-actin).
